# Molecular Dating of the Teleost Whole Genome Duplication (3R) Is Compatible With the Expectations of Delayed Rediploidization

**DOI:** 10.1093/gbe/evae128

**Published:** 2024-06-24

**Authors:** Minbo Qi, James Clark, Edmund R R Moody, Davide Pisani, Philip C J Donoghue

**Affiliations:** Bristol Palaeobiology Group, School of Biological Sciences, University of Bristol, Life Sciences Building, Bristol BS8 1TQ, UK; Bristol Palaeobiology Group, School of Biological Sciences, University of Bristol, Life Sciences Building, Bristol BS8 1TQ, UK; Bristol Palaeobiology Group, School of Earth Sciences, University of Bristol, Life Sciences Building, Bristol BS8 1TQ, UK; Bristol Palaeobiology Group, School of Biological Sciences, University of Bristol, Life Sciences Building, Bristol BS8 1TQ, UK; Bristol Palaeobiology Group, School of Earth Sciences, University of Bristol, Life Sciences Building, Bristol BS8 1TQ, UK; Bristol Palaeobiology Group, School of Earth Sciences, University of Bristol, Life Sciences Building, Bristol BS8 1TQ, UK

**Keywords:** whole genome duplication, molecular clock dating, teleost diversification, delayed rediploidization

## Abstract

Vertebrate evolution has been punctuated by three whole genome duplication events that have been implicated causally in phenotypic evolution, from the origin of phenotypic novelties to explosive diversification. Arguably, the most dramatic of these is the 3R whole genome duplication event associated with the origin of teleost fishes which comprise more than half of all living vertebrate species. However, tests of a causal relationship between whole genome duplication and teleost diversification have proven difficult due to the challenge of establishing the timing of these phenomena. Here we show, based on molecular clock dating of concatenated gene alignments, that the 3R whole genome duplication event occurred in the early–middle Permian (286.18 to 267.20 million years ago; Ma), 52.02 to 12.84 million years (Myr) before the divergence of crown-teleosts in the latest Permian–earliest Late Triassic (254.36 to 234.16 Ma) and long before the major pulses of teleost diversification in Ostariophysi and Percomorpha (56.37 to 100.17 Myr and at least 139.24 to 183.29 Myr later, respectively). The extent of this temporal gap between putative cause and effect precludes 3R as a deterministic driver of teleost diversification. However, these age constraints remain compatible with the expectations of a prolonged rediploidization process following whole genome duplication which, through the effects of chromosome rearrangement and gene loss, remains a viable mechanism to explain the evolution of teleost novelties and diversification.

**Significance**

Teleost fishes are a hugely diverse vertebrate group, and their origin is associated with a genome duplication event that is commonly thought causal to teleost diversification. We attempted to test this hypothesis by estimating the absolute timing of genome duplication and speciation events, showing that genome duplication occurred tens of millions of years before teleost diversification. This is incompatible with a direct causal relationship but compatible with the expectations of the prolonged period required for chromosome pairing to stabilize, only after which large scale genetic divergence and its phenotypic consequences become possible. This may be a general expectation for the outcome of genome duplication events.

## Introduction

Whole genome duplications (WGDs) have occurred in many species-rich lineages (e.g. arthropods (Schwager et al. 2017; Li et al. 2018), plants (Jiao et al. 2011; Li et al. 2015), fungi (Scannell et al. 2006; Albertin and Marullo 2012), and vertebrates (Dehal and Boore 2005; Ohno 1970; Glasauer and Neuhauss 2014). This process creates functionally redundant gene copies that are thought to be released from selective pressure, becoming free to either lose or gain functions, leading to evolutionary innovation (Ohno 1970); Glasauer and Neuhauss 2014; Soltis and Soltis 2016; Jiao 2018; Landis et al. 2018). Three WGD events have been proposed to have played a causal role in vertebrate evolutionary history. The 1R and 2R events occurred very early in vertebrate evolution and have been associated with major phenotypic and embryological innovations (Shimeld and Holland 2000; Ohno 1970; Yu et al. 2024). The 3R event is associated with the hyperdiverse teleosts (Glasauer and Neuhauss 2014), and it is considered a model for exploring the role of WGD in organismal evolution. This is because 3R occurred recently enough for its effects on the genome to still be visible, while being ancient enough for its evolutionary potential to have been realized (Stellwag 2004). Indeed, the fate of the genes in the genomes of teleost lineages postdating the 3R event has been thoroughly studied (Parey et al. 2020, 2022, 2023). However, it has proven difficult to precisely test what processes in teleost evolution the 3R event precipitated, and whether it played a causal role in facilitating the emergence of the teleost diversity, because of a lack of accuracy and precision in the timing of the 3R event. Indeed, previous attempts to constrain the 3R event using molecular clocks have yielded a broad range of estimates spanning from 400 to 226 Ma (387 to 253 Ma (Vandepoele et al. 2004); ∼350 Ma (Christoffels et al. 2004); 380 to 400 Ma (Christoffels et al. 2006); 316 to 226 Ma (Hurley et al. 2007)), while fossil-based inference of genome size from osteocyte lacunae has provided a minimum constraint of 235 Ma (Davesne et al. 2021). Here we attempt to better constrain the timing of the 3R event to facilitate effective tests of its causal consequences. To achieve this goal, we leverage greater precision through molecular clock analysis of concatenated ohnologues (paralogues originating from WGD events; for simplicity, we refer to orthogroups including ohnologue pair simply as “ohnologue pairs”; Wolfe 2000). We find that the 3R event can be constrained to the interval 285.8 to 266.77 Ma (early to mid-Permian). We explore the implications of our estimated divergence times on the origin of crown-teleosts, including on previous findings about the process of dysploidy invoked by the dosage balance hypothesis as the motor of evolutionary innovation following WGD (Hughes et al. 2007; Konrad et al. 2011; Conant et al. 2014). There can be no doubt that WGD contributed to the evolution of clades such as teleosts since 3R ohnologues are utilized in the development of teleost innovations (Glasauer and Neuhauss 2014; Moriyama and Koshiba-Takeuchi 2018). However, it does not follow that these innovations could not have evolved without WGD. Therefore, we question whether there is a direct deterministic and immediate relationship between WGD and evolutionary innovation—in this instance and, perhaps, in general.

## Materials and Methods

Molecular clock methods are usually applied to species trees to infer the timing of species divergences; however, they can just as readily be applied to gene trees to estimate the timing of both species’ divergences and gene duplication events. When dating whole genome duplication events, it is possible to achieve greater precision through concatenating ohnologue pairs (Macqueen and Johnston 2014; Clark and Donoghue 2017, 2023).

**Fig. 1.**
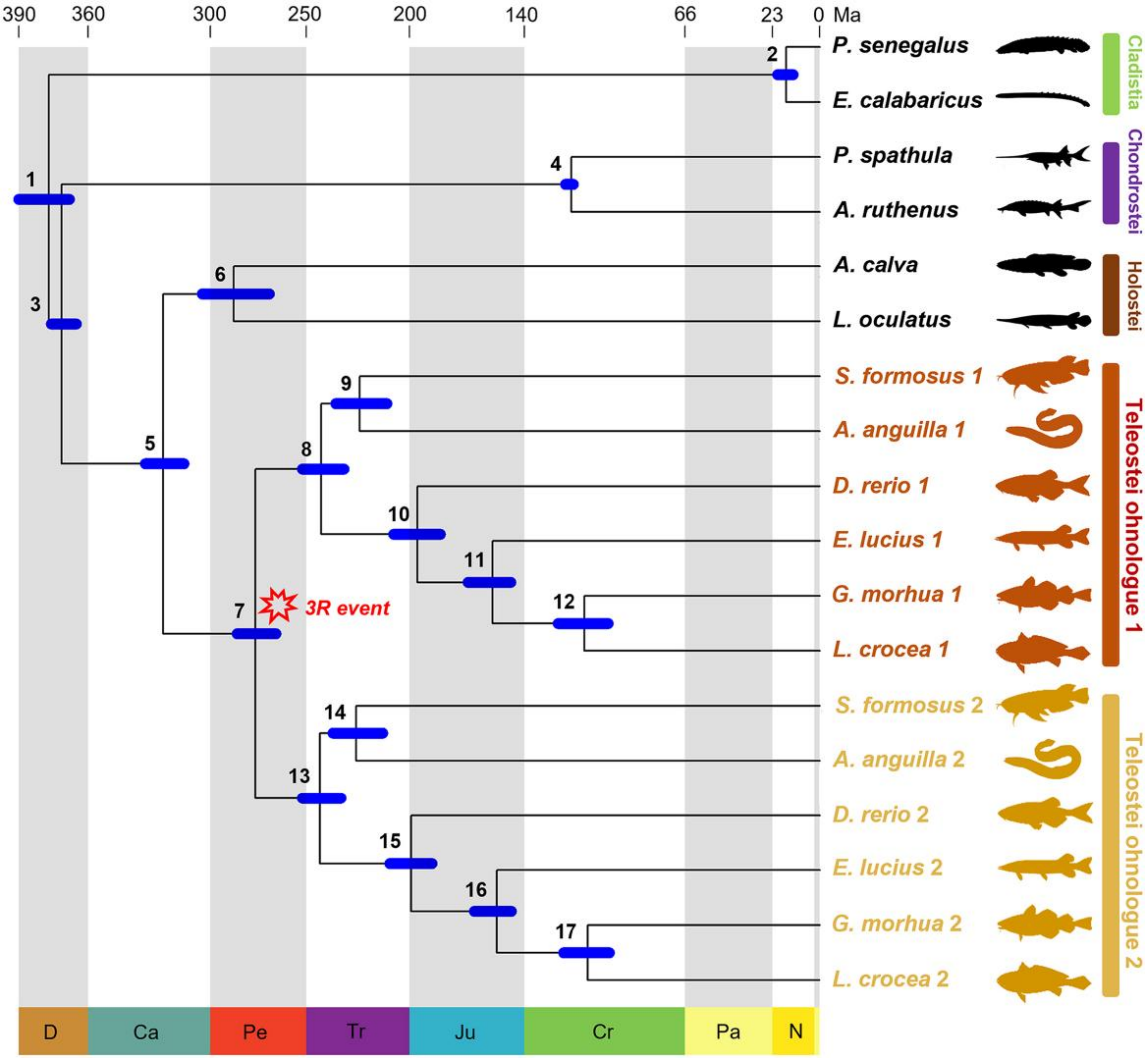
Divergence time estimates for the 3R WGD event (node 7) and the actinopterygian lineage divergences based on a concatenation of 30 ohnologue pairs. Age estimates represent the 95% highest posterior density (HPD). The two teleost ohnologue groups are distinguished by different colors (orange and burnt orange). Node numbers represent the following clades: node 1: crown Actinopterygii; node 2: crown Cladistia; node 3: crown Actinopteri; node 4: crown Chondrostei; node 5: crown Neopterygii; node 6: crown Holostei; node 7: 3R; node 8 and node 13: crown Teleostei; node 9 and node 14: divergence of Osteoglossomorpha and Elopocephalai; node 10 and node 15: crown Clupeocephala; node 11 and node 16: divergence of Protacanthopterygii and (Neoteleostei + Stomiati); node 12 and node 17: crown Acanthomorpha; abbreviations for geological periods: D: Devonian; Ca: Carboniferous; Pe: Permian; Tr: Triassic; Ju: Jurassic; Cr: Cretaceous; Pa: Paleogene; N: in short for Neogene.

To build the dataset for our molecular clock analysis, we sampled 12 species representing the major lineages of ray-finned fish; the protein sequences were obtained from the genome assembly of each selected species at NCBI (supplementary table S7, Supplementary Material online, only genomes with proteome available were selected). We used cdhit v4.8.1 (Li and Godzik 2006; Fu et al. 2012) to remove redundant isoforms from each genome assembly, and orthogroups (the set of genes from multiple species descended from a single gene in the last common ancestor of that set of species) containing ohnologues were identified with OrthoFinder v2.3.7 (Emms and Kelly 2015, 2019) using DIAMOND (Buchfink et al. 2021). Orthogroups were selected through visual inspection of gene trees on the basis that they conformed to ohnologue divergence prior to the crown-teleost speciation event (Fig. 1; (Robertson et al. 2017)). To ensure uniformity across selected orthogroups (we refer these orthogroups, each containing a pair of ohnologues from each of the six teleost species and non-duplicated genes from six non-teleost species simply as “ohnologue pairs”), we removed highly similar isoforms and duplications unrelated to 3R (note that the removal of these sequences did not affect the data available for selecting teleost 3R ohnologues since these gene copies are in the same homology relationships with genes from the 3R, so keeping one is enough). Sequences were aligned using MAFFT v7.429 (Katoh and Standley 2013) and TrimAl v1.2 (Capella-Gutiérrez et al. 2009); model selection was performed using ModelFinder (Kalyaanamoorthy et al. 2017) in IQ-TREEv1.6.12 (Nguyen et al. 2015; Chernomor et al. 2016). Ohnologues pairs with sequence alignments shorter than 100 amino acids were discarded. This resulted in the final dataset containing a total of 30 ohnologue pairs (27 of which preserved all anticipated genes in all species) with 17286 amino acids in 537 sequences (see supplementary tables S5, S6, and S8, Supplementary Material online for detailed information on each sequence, ohnologue, and dataset). We performed KEGG pathways enrichment analyses through the WebGestalt web-server (Liao et al. 2019) to ensure the absence of sampling bias in our dataset. Datasets with different sizes (with 1, 6, 12, and 18 ohnologue pairs concatenated) for infinite site plot analyses were built by sequentially concatenating ohnologue pairs sampled at random from our largest dataset of 30 ohnologue pairs. To be more specific, we started with one random ohnologue pair, added five to generate the dataset of six ohnologue pairs, then added another 6 to generate the dataset of 12 ohnologue pairs, and finally added another 6 for the dataset including 18 ohnologue pairs. Ohnologue pairs were dated individually, and as a single concatenated alignment where they were treated as separate partitions (in datasets) that were modeled using the best-fitting amino acid substitution models (see supplementary table S9, Supplementary Material online for detailed information), identified using the Bayesian Information Criterion. The species phylogeny used to date duplicated genes was constrained to conform to the Actinopterygian phylogeny in Dornburg and Near (2021).

**Fig. 2.**
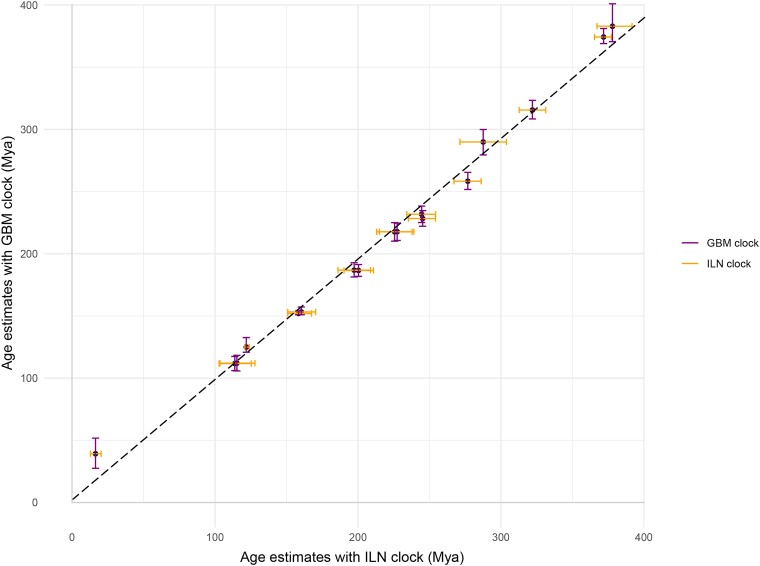
Comparison of age estimates based on the ILN clock model (orange) and the GBM clock model (purple), with error bar showing the 95% HPD intervals.

Speciation node ages in the tree were constrained with fossil calibrations comprising hard minima and soft maxima, formulated following best practice (Parham et al. 2011), many revised from Benton et al. (2015) (see Supplementary material section 3). The prior probability of divergence times was modeled using a uniform distribution (97.5%), reflecting equal probability per unit time between hard minimum and soft maximum bounds, supplemented with a 2.5% tail distribution extending from the maximum constraint, representing the low (but nonzero) probability that the true age exceeds the soft maximum. We used the normal approximation method of MCMCtree in PAML 4.9h (Rannala and Yang 2007; dos Reis and Yang 2011) for divergence time estimation. That is, we estimated the branch length information for each ohnologue pair with the best-fitting amino acid substitution models (in codeml, see supplementary table S9, Supplementary Material online) and employed a gamma distribution (“rgene_gamma” setting in the mcmctree control file) to model the variation of evolutionary rate across ohnologue pairs (see supplementary table S10, Supplementary Material online). The branch lengths were then used to estimate divergence times in further single-gene and concatenated analyses. For the concatenated analyses, each ohnologue pair was treated as one partition. In each analysis, the sigma2_gamma was set to alpha = 1 and beta = 10, the prior birth–death process was set with parameters of birth = 1, death = 1, and sampling rate = 0.1. We explored the impact of using different clock models (ILN and GBM) to determine the sensitivity of timescale estimation to assumption of rate heritability (Fig. 2). Each analysis was repeated twice, and convergence assessed using Tracer v. 1.7 (Rambaut et al. 2018), while infinite site plots and violin plots were composed using Statistical Software R (v3.5.2; R Core Team 2023 ).

## Results

The evolutionary timescale derived from our main analysis of the concatenated alignment of ohnologue pairs using the independent rates (ILN) clock model estimates the divergence of crown Actinopterygii at 391.70 to 367.22 Ma (Middle–Late Devonian), crown Actinopteri at 377.37 to 365.46 Ma (Late Devonian), Neopterygii at 331.33 to 312.76 Ma (Middle Mississippian to Middle Pennsylvanian), and crown Teleostei at 254.36 to 234.16 Ma (late Permian to earliest late Triassic). The divergence of Elopomorpha and Osteoglossomorpha is dated to 239.12 to 213.05 Ma (Middle–Late Triassic), Clupeocephala at 210.83 to 186.01 Ma (Late Triassic–Early Jurassic), divergence of Protacanthopterygii (Pike) from other teleosts at 170.41 to 150.94 Ma (Middle–Late Jurassic), Acanthomorpha at 127.96 to 102.89 Ma (Early Cretaceous), Holostei at 303.82 to 271.33 Ma (Late Pennsylvanian to middle Permian), Chondrostei at 124.02 to 120.80 Ma (Early Cretaceous), and Cladistia at 20.37 to 12.94 Ma (Early–Middle Miocene) (Fig. 1 and supplementary table S1, Supplementary Material online). Analysis of the concatenation using the geometric Brownian motion (GBM) clock model yielded highly consistent results. Regression analysis suggests that the results from the ILN clock are generally older but a two-tailed *t*-test indicates that there is no significant difference between the two results (*P* ∼0.07) (Fig. 2 and supplementary table S1, Supplementary Material online). Accordingly, we mostly report the ILN results, which we compare against the GBM results as necessary.

**Fig. 3.**
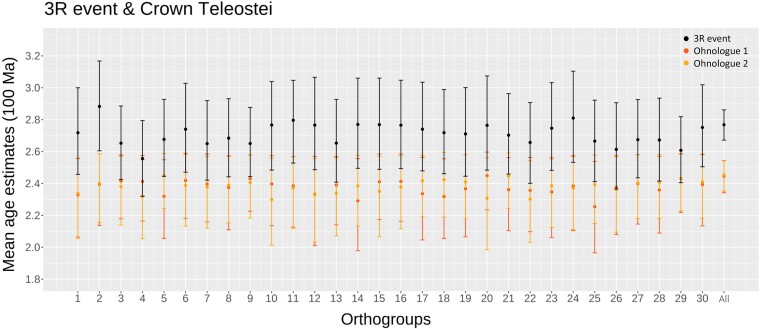
Age estimates of 3R event and the divergence of crown teleost based on different ohnologue pairs. The numbers correspond to individual ohnologue pair, while “all” represents the results obtained from the concatenated dataset comprising all 30 ohnologue pairs. The central dot within each data represents the mean age estimate, while the surrounding bars depict the 95% HPD interval of the estimate. Please refer to the supplementary fig. S1, Supplementary Material online for age estimates of other nodes.

**Fig. 4.**
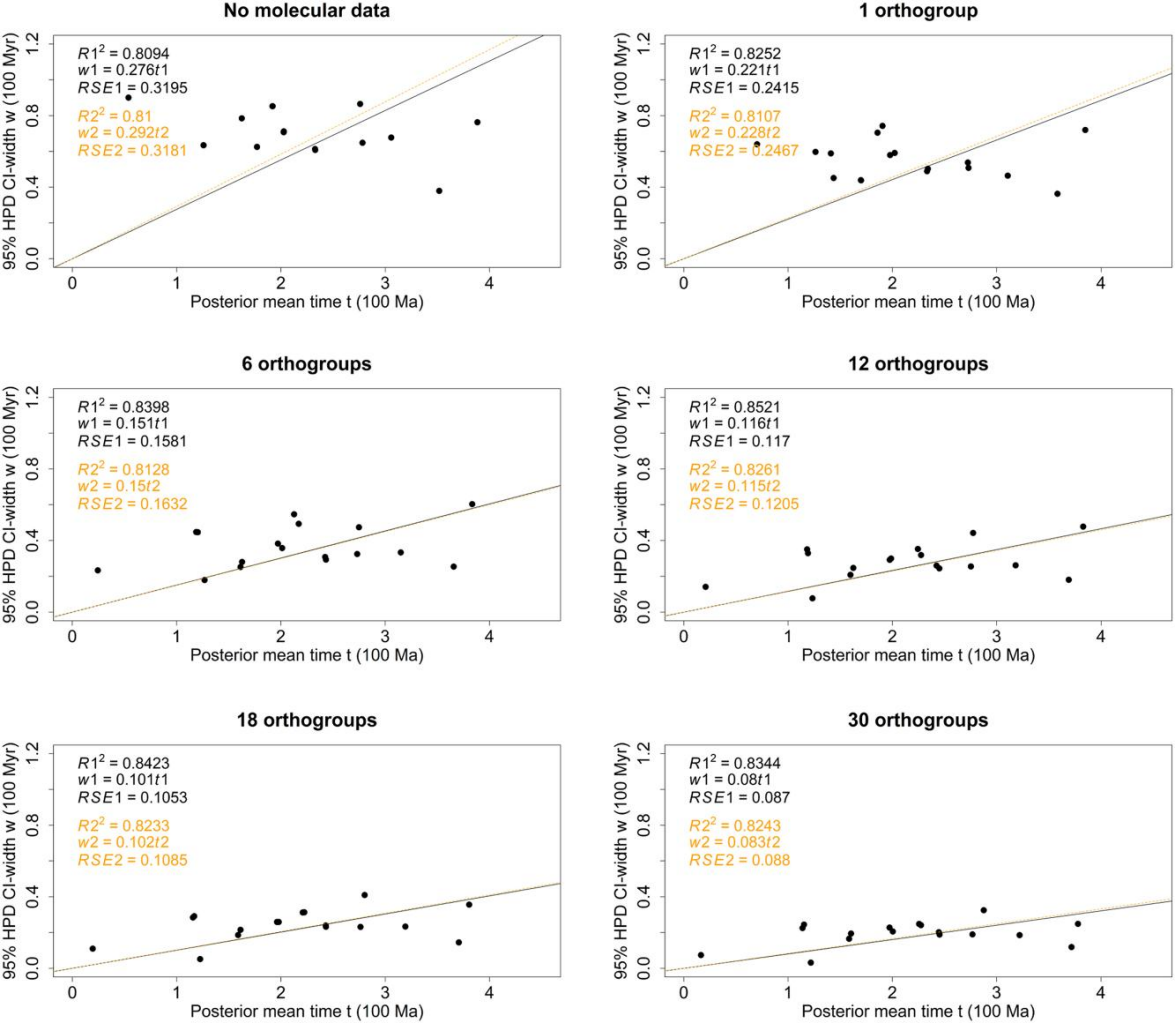
Infinite sites plots showing the estimated posterior mean time in 100 Ma (*x* axis) plotted against the estimated 95% HPD interval widths in 100 Myr (*y* axis) for the dating analyses using dataset with different number of ohnologue pairs (orthogroups) concatenated (no molecular data refers to analyses run with calibrations and a tree prior but no sequence data, while 1, 6, 12, 18, and 30 ohnologue pairs refer to the size of datasets used in each analysis). The solid black line and dotted orange line represent the regression line including the root node and excluding the root node, respectively. The *R*^2^ (coefficient of determination), residual standard error (RSE), and the equations of the regression lines with (black) and without (orange) the root are shown.

**Fig. 5.**
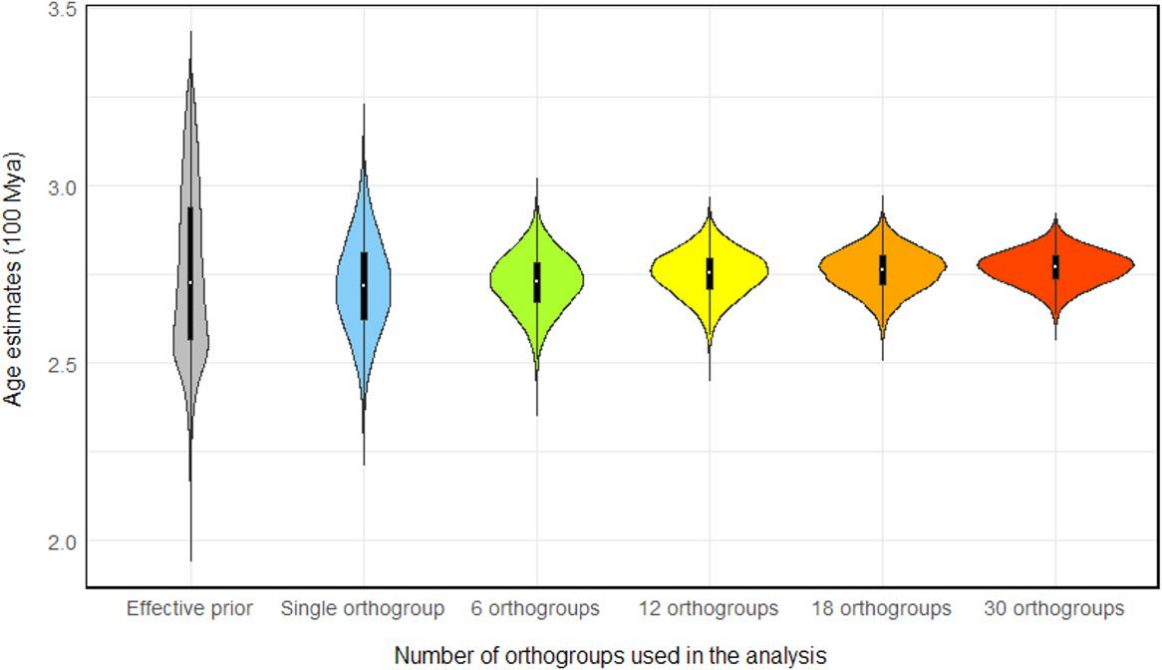
Posterior distributions for the age of the 3R event when datasets concatenating different numbers of ohnologue pairs (orthogroups) are analyzed.

Analyses of individual ohnologue pair using the ILN clock model produced comparable results to the concatenation of all ohnologue pairs, but at lower precision. Although some variation exists in both the mean age estimates and the 95% highest posterior density (HPD) interval width across different ohnologue pairs, the results consistently overlap and demonstrate congruence with each other (Fig. 3). We used infinite sites plots to assess the impact of adding molecular data on precision of divergence time estimates, with results from analysis performed without sequence data (the effective priors) serving for comparison (Fig. 4). For all nodes in the tree, the 95% HPD for the node age estimate diminishes dramatically as the sequence alignment increases in size. The *R*^2^ score in all regression analyses was ∼0.85 (as shown in Fig. 4) with the standard error of the residual (*σ*) reducing through 0.3195, 0.2415, 0.1581, 0.117, 0.1053, and 0.087 for analyses performed without data or based on progressively larger datasets of 1, 6, 12, 18, and 30 ohnologue pairs (respectively). Furthermore, with the inclusion of additional sequence data, the slope of the regression model in the infinite site plot shows a significant and pronounced reduction, declining from 0.276 (in the absence of data) to 0.08 (when considering 30 ohnologue pairs), indicating that in the estimation of our largest dataset, for every 1 Myr of divergence, 0.08 Myr of uncertainty is added to the 95% HPD. A violin plot (Fig. 5) illustrates the effective prior distribution and posterior distributions of node 3R with different numbers of ohnologue pairs used.

The timing of the 3R event is estimated (using the ILN clock) at 286.18 to 267.20 Ma (Artinskian–Roadian; early–middle Permian) with an uncertainty of just 18.98 Myr (Figs. 1 and 5 and supplementary table S2, Supplementary Material online). Using the GBM clock, the 3R age estimate is slightly younger, at 265.45 to 251.68 Ma (Wordian–Induan; middle Permian to earliest Triassic).

## Discussion

### Timescale of Species Divergences

Our estimates for the species divergence times are consistent between ILN and GBM clock models. While estimates based on individual genes exhibit some variance, there is considerable overlap between estimates obtained from different genes or using the full, concatenated dataset. Our infinite sites plots, based on different numbers of concatenated ohnologue pairs (0 to 30), demonstrate that the addition of sequence data leads to significant increases in precision, and this shows no sign of reaching an asymptote in our largest concatenation of 30 ohnologue pairs. This suggests that more precision could be leveraged by adding additional ohnologue pairs but, while there are still thousands of additional ohnologue pairs that could be considered (Parey et al. 2022), it is challenging to find more candidates where the gene trees are compatible with species relationships and the anticipated pattern of paralogy arising from the 3R event. Thus, while it may be possible, through the addition of more sequence data, to achieve greater precision for the divergences of the species in our dataset, we argue that our divergence time estimates for the 3R event are close to the maximal level of precision that can be achieved while being confident that the considered genes are indeed dating the 3R event. We performed further analyses to make sure that our selected genes are ohnologues and that they have a broad physical and functional distribution (supplementary tables S3 to S6, Supplementary Material online). This is important to ensure that we are not dating the history of just one part of the genome, or of genes with a specific history that is distinct from that of the rest of the genome. Based on zebrafish sequences, we mapped our ohnologues (see supplementary tables S5 and S6, Supplementary Material online for their accession numbers and Ensembl gene ID) on the FishComparativeAtlas database (Parey et al. 2022), finding that all of our orthologues with available Ensembl IDs are evenly distributed on different chromosomes, and that there is no significant functional enrichment for our set of genes (see supplementary table S6, Supplementary Material online for their location on modern and ancestral chromosomes and supplementary tables S3 and S4, Supplementary Material online for the result of enrichment analysis). These results confirm that the genes in our dataset represent a broad sample of the actinopterygian genomes considered, unbiased in terms of synteny of function and, therefore, representative of its evolutionary history.

Our species divergence time estimates are broadly comparable to recent studies. The estimates for the clades Actinopterygii (391.70 to 367.22 Ma), Actinopteri (377.37 to 365.46 Ma), Neopterygii (331.33 to 312.76 Ma), Chondrostei (124.02 to 120.80 Ma), Teleostei (254.36 to 234.16 Ma), and Clupeocephala (210.83 to 186.01 Ma) are consistent with those of Hughes et al. (2018) (Actinopterygii: 379 Ma; Actinopteri: 350 Ma; Neopterygii: 295 Ma; Teleostei: 250 Ma, respectively) and Redmond et al. (2023) (Actinopterygii: 384 to 372.1 Ma; Actinopteri: 374.8 to 360.6 Ma; Neopterygii: 319.9 to 270.8 Ma; Holostei: 306.4 to 251.4 Ma; Chondrostei: 124.1 to 203.3 Ma; Clupeocephala: 149.2 to 202.2 Ma), though our estimates for Actinopteri and Neopterygii are slightly older. In large part, these similarities and minor differences are driven by similarities and differences in the calibrations used and the taxon set chosen to address the different questions these studies focus upon. Nevertheless, the overwhelming similarity in these timescales, despite their different approaches and sequence datasets, provides confidence in our evolutionary timescale and its implications for the timing of the 3R event.

Our clade age estimates are inevitably compatible with the fossil record since the analyses were constrained by fossil-based clade-age minima. Nevertheless, it may be worth reflecting on the plausibility of the inferred time span between clade age estimates and fossil minima. Davesne et al. (2021) reviewed the fossil record of teleosts and their relatives, concluding that there are no unambiguous total-group teleosts before the Triassic, whereas we infer Neopterygii diverging in the Late Mississippian–Early Pennsylvanian, and no convincing crown-teleosts before the Middle Jurassic, whereas we infer a latest Permian–late Triassic origin. However, we should not anticipate molecular estimates of clade age to closely approximate fossil minima since lineage divergence is a genomic, not a phenotypic phenomenon (Donoghue and Yang 2016). Thus, while early crown-teleosts would have been genomically divergent, they would have been anatomically indistinguishable from one another and from stem teleosts, until their descendents evolved anatomical novelties that could be preserved in the fossil record. Furthermore, the early fossil record of neopterygians is self-evidently incomplete since crown Holostei are known from the earliest Triassic (Friedman 2022), requiring their sibling lineage, total-group teleosts, to be at least as old. Furthermore, the marine fossil record of actinopterygians through the Pennsylvanian–middle Permian is particularly poorly sampled (Henderson et al. 2023). For these reasons, we see no concern in the inferred time span between our clade age estimates and fossil minima.

### Molecular Dating Suggests an Early–Middle Permian 3R

Our dating of the concatenated ohnologue pairs estimated 3R to have occurred in the interval 286.18 to 267.20 Ma (early–middle Permian), with an attendant uncertainty of 18.98 Myr. This result is consistent with, but considerably more precise than previous studies that have estimated 3R to have occurred 316 to 226 Ma using a relaxed molecular clock (Hurley et al. 2007) or >235 Ma based on an analysis of bone cell lacunae in stem teleosts (Davesne et al. 2021). However, our results are inconsistent with some previous divergence time studies that estimated 3R to have occurred >300 Ma and 400 to 320 Ma (Christoffels et al. 2004; Vandepoele et al. 2004; Christoffels et al. 2006), but these were based on out-moded strict molecular clock methods that do not account for the effect of rate variation across lineages (Ho and Duchêne 2014).

In dating 3R, it is important we reflect on the event or episode we have dated within the processes of whole genome duplication and rediploidization. Most previous attempts to date 3R have assumed, perhaps implicitly, that descendent ohnologues begin to diverge immediately. However, in well-studied WGD events associated with salmon and chondrostean fishes, the genome of new autopolyploids underwent a prolonged period of tetrasomic inheritance during which duplicated chromosomes formed tetravalent pairing during meiosis (Robertson et al. 2017; Parey et al. 2022; Redmond et al. 2023). Parey et al. (2022) have shown that this obtained in association with the 3R event, too.

Homologous recombination processes (e.g. gene conversion) prohibit the divergence of ohnologues (Otto 2007; Lien et al. 2016; Redmond et al. 2023) until sufficient sequence differences have accumulated among homologous chromosomes and the genome gradually transits back to disomic inheritance (Parey et al. 2022; Redmond et al. 2023). When homologous chromosomes begin to follow disomic inheritance, they have accumulated enough differences to behave as two pairs of distinct chromosomes during meiosis. Subsequently, ohnologue pairs derived from WGD can diverge uninterruptedly (Otto 2007).

Given this, our estimate for 3R may represent only the end of the tetrasomic inheritance period and, therefore, provide only a minimum constraint on the timing of the 3R event according to previous research (Parey et al. 2022; Redmond et al. 2023). However, Node 3R represents the coalescence point at which ohnologues trace back to their ancestral state, the point at which ohnologue sequences are identical. Thus, the divergence between ohnologue sequences occurs, but it is inhibited and obscured by recombination and gene conversion during tetrasomic inheritance, as seen in previous studies showing differences between tetrasomically inherited ohnologues (Campbell et al. 2019; Parey et al. 2022). Additionally, homologous recombination processes not only suppress ohnologue divergence but also disseminate mutations across all ohnologues, facilitating the divergence of ohnologues from their shared ancestral state as soon as the WGD event occurred. Consequently, we interpret the age estimate of Node 3R as corresponding to the 3R event itself, when two duplicates of the ancestral genome emerge and start to diverge from the ancestral genome, rather than the time point when tetrasomic inheritance ends and the two ohnologues start to diverge uninhibitedly from each other.

There can be no doubt that the rate of divergence would vary between tetrasomic and disomic inheritance; it is not realistic to incorporate this rate variance into current divergence time analysis since it is so poorly understood. Nevertheless, the point of divergence remains the WGD event itself, not the end of the period of tetrasomic inheritance, and failure to correctly model rate variation before and after disomic inheritance is likely to bias estimates for the timing of 3R. However, we anticipate that our approach of dating 3R based on a concatenation of ohnologue pairs (derived from a variety of different ancestral chromosomes) that diverged at slightly different times and rates will have an averaging effect that will mitigate against this bias. We conclude that our results identify the timing of the 3R event itself.

### The Role of WGD in Phenotypic Evolution: 3R and Beyond

Since its discovery (Amores et al. 1998), the 3R WGD event has been invoked as a causal factor in the diversification of teleost fishes, though material links to innovation and diversification have not been well defined (Ravi and Venkatesh 2008; Glasauer and Neuhauss 2014; Moriyama and Koshiba-Takeuchi 2018). As with previous attempts to constrain its timing (Christoffels et al. 2004; Vandepoele et al. 2004; Christoffels et al. 2006; Hurley et al. 2007; Davesne et al. 2021), we find that 3R occurred before (12.84 to 52.02 Myr) the diversification of crown-teleosts. However, much of teleost diversity stems not from the crown-ancestor, but from the ostariophysan and percomorph clades (Donoghue and Purnell 2005; Hurley et al. 2007; Santini et al. 2009; Davesne et al. 2021), and our estimate of 3R is even more remote from these secondary diversification events (a time lag of 56.37 to 100.17 Myr for crown ostariophysan and at least 139.24 to 183.29 Myr for crown Percomorpha, respectively) than from the primary diversification event at the teleost crown clade. A similar temporal and phylogenetic disconnect occurs between 3R and the phenotypic innovations that have been attributed to it, like electric organs and the switch of visual pigment expression during migration (Glasauer and Neuhauss 2014; Nakamura et al. 2017); functional innovations invoked to explain teleost diversification are just as remote (Davesne et al. 2021). This may be interpreted to reject a causal relationship between 3R and teleost diversification (Donoghue and Purnell 2005; Davesne et al. 2021). However, the extensive time spans between proposed cause and effect are consistent with post-WGD rediploidization processes (facilitated by major genomic reorganizations such as chromosome fusions, fissions, deletions, or inversions) (Inoue et al. 2015) which are likely the real drivers of the evolutionary change arising from WGD (Robertson et al. 2017). The Lineage-Specific Ohnologue Resolution model has been proposed to explain how this phenomenon of delayed rediploidization can drive species radiation tens of millions of years after WGD (Robertson et al. 2017). Indeed, delayed rediploidization of ohnologues is common in autopolyploidy WGD events. For example, in salmonids, approximately one-quarter of the duplicated genome is estimated to remain unrediploidized >50 Myr after Ss4R WGD (Lien et al. 2016; Robertson et al. 2017; Campbell et al. 2019). Similarly, within Acipenseriformes, 50% to 66% of the genome remains unresolved ∼80 Myr after WGD (Redmond et al. 2023). Recent research also uncovered evidence indicating that entire chromosomes experienced delayed rediploidization in teleost for at least 60 Myr after 3R, which aligned well with our time estimation (Parey et al. 2022). Evidently, a delay between WGD and any phenotypic consequences should be an expectation (for autopolyploidy events at least) and this appears to be a general pattern, as seen in land plants (Eric Schranz et al. 2012; Clark and Donoghue 2017, 2018), among other clades.

This makes the test of hypotheses of causality challenging, since it becomes difficult to discriminate innovations that arose from WGD. It may not be enough to demonstrate that an ohnologue is implicated in the emergence of an evolutionary novelty (e.g. Moriyama and Koshiba-Takeuchi (2018); Peskin et al. (2020)) since the effects of a single gene are not qualitatively or quantitatively different from the effects of tandem gene duplication. To be sure, WGD events engender much greater evolvability since they generate a genome's worth of gene duplicates available for innovation, but this is really only an endmember within a spectrum of effects that range through chromosomal and segmental duplications, widespread gene duplication, to tandem duplication of a single gene. The anticipated effects in terms of evolvability should be commensurate with the magnitude of these genomic changes. More than anything, however, we should anticipate that the impact of WGD on phenotypic evolution should be more permissive and protracted, as suggested by our dating result and emerging evidence from other recent research (Robertson et al. 2017; Parey et al. 2022), rather than deterministic and immediate, as proposed in early interpretations of the three main vertebrate WGD events (e.g. Ruddle, et al. (1994a); Ruddle, et al. (1994b); Sidow (1996); Stellwag (1999)). This is because post-WGD ohnologue retention is not random, with developmental genes and transcription factors that are important in phenotypic determination being preferentially retained, in association with expanded repertoires of regulatory elements (Marlétaz et al. 2018). This is the impact on teleost evolution that 3R has had, the legacy of which continues to play out.

## Supplementary Material

evae128_Supplementary_Data

## Data Availability

The information of datasets (alignments and calibrated tree) used in our research are provided in the electronic Supplementary material, and data can be downloaded from the GitHub website (https://github.com/Rionay1/Raw_data_for_Ts3R_research.git).
