# Supplementary material for: Molecular Dating of the Teleost Whole Genome Duplication (3R) Is Compatible With the Expectations of Delayed Rediploidization
Source: Genome Biol Evol. 2024 Jun 24;16(7):evae128. doi: 10.1093/gbe/evae128 (PMC11259977; doi:10.1093/gbe/evae128)
Supplement: evae128_Supplementary_Data [file evae128_supplementary_data.zip › 3_Supplementary_Table_5_sequence_ID.pdf]

Orthogroup	Common name	Species name (abbreviation)	Sequence ID
	Senegal bichir	P_senegalus	XP_039595471.1
	Reedfish	E_calabaricus	XP_028674418.1
	Sterlet	A_ruthenus	XP_033879666.1
	American paddlefish	P_spathula	XP_041090021.1
	Bowfin	A_calva	MBN3310886.1
	Spotted gar	L_oculatus	XP_006631328.1
	European eel_1	A_anguilla_1	XP_035287501.1
	Asian arowana_1	S_formosus_1	XP_018610315.1
Orthogroup 1	Zebrafish_1	D_rerio_1	XP_009290780.1
	Northern pike_1	E_lucius_1	XP_012988282.1
	Atlantic cod_1	G_morhua_1	XP_030226940.1
	Large yellow croaker_1	L_crocea_1	XP_010752699.2
	European eel_2	A_anguilla_2	XP_035286632.1
	Asian arowana_2	S_formosus_2	XP_018603299.2
	Zebrafish_2	D_rerio_2	NP_001038647.1
	Northern pike_2	E_lucius_2	XP_034150766.1
	Atlantic cod_2	G_morhua_2	XP_030203117.1
	Large yellow croaker_2	L_crocea_2	XP_010751943.3
	Senegal bichir	P_senegalus	XP_039624404.1
	Reedfish	E_calabaricus	XP_028654705.1
	Sterlet	A_ruthenus	XP_034775767.1
	American paddlefish	P_spathula	XP_041089477.1
	Bowfin	A_calva	MBN3296910.1
	Spotted gar	L_oculatus	XP_006625629.1
	European eel_1	A_anguilla_1	XP_035235109.1
	Asian arowana_1	S_formosus_1	XP_018599645.1
Orthogroup 2	Zebrafish_1	D_rerio_1	NP_001074095.1
	Northern pike_1	E_lucius_1	XP_010901136.1
	Atlantic cod_1	G_morhua_1	XP_030227149.1
	Large yellow croaker_1	L_crocea_1	XP_010752263.1
	European eel_2	A_anguilla_2	XP_035246685.1
	Asian arowana_2	S_formosus_2	XP_018608184.2
	Zebrafish_2	D_rerio_2	NP_001092714.1
	Northern pike_2	E_lucius_2	XP_010879651.1
	Atlantic cod_2	G_morhua_2	XP_030230299.1
	Large yellow croaker_2	L_crocea_2	XP_010740435.2
	Senegal bichir	P_senegalus	XP_039608714.1
	Reedfish	E_calabaricus	XP_028658931.1
	Sterlet	A_ruthenus	XP_033869603.1
	American paddlefish	P_spathula	XP_041085427.1
	Bowfin	A_calva	MBN3310257.1
	Spotted gar	L_oculatus	XP_006630105.1
	European eel_1	A_anguilla_1	XP_035283049.1
	Asian arowana_1	S_formosus_1	XP_018601741.2
Orthogroup 3	Zebrafish_1	D_rerio_1	XP_001338964.6
	Northern pike_1	E_lucius_1	XP_012989032.1
	Atlantic cod_1	G_morhua_1	XP_030224850.1
	Large yellow croaker_1	L_crocea_1	XP_019109297.1
	European eel_2	A_anguilla_2	XP_035274581.1
	Asian arowana_2	S_formosus_2	XP_018591185.2
	Zebrafish_2	D_rerio_2	XP_021326868.1
	Northern pike_2	E_lucius_2	XP_034146905.1
	Atlantic cod_2	G_morhua_2	XP_030207087.1
	Large yellow croaker_2	L_crocea_2	XP_019121740.1
	Senegal bichir	P_senegalus	XP_039603624.1
	Reedfish	E_calabaricus	XP_028653602.1
	Sterlet	A_ruthenus	XP_033859897.1
	American paddlefish	P_spathula	XP_041106451.1
	Bowfin	A_calva	MBN3310624.1

Orthogroup 4	Spotted gar	L_oculatus	XP_015213153.1
	European eel_1	A_anguilla_1	XP_035261673.1
	Asian arowana_1	S_formosus_1	XP_018585919.1
	Zebrafish_1	D_rerio_1	XP_002665313.2
	Northern pike_1	E_lucius_1	XP_010877425.1
	Atlantic cod_1	G_morhua_1	XP_030213235.1
	Large yellow croaker_1	L_crocea_1	XP_010742105.3
	European eel_2	A_anguilla_2	XP_035276588.1
	Asian arowana_2	S_formosus_2	XP_018599013.1
	Zebrafish_2	D_rerio_2	NP_001093461.1
	Northern pike_2	E_lucius_2	XP_010893323.3
	Atlantic cod_2	G_morhua_2	XP_030201078.1
	Large yellow croaker_2	L_crocea_2	XP_027135104.1
Orthogroup 5	Senegal bichir	P_senegalus	XP_039619912.1
	Reedfish	E_calabaricus	XP_028666117.1
	Sterlet	A_ruthenus	XP_034768977.1
	American paddlefish	P_spathula	XP_041098227.1
	Bowfin	A_calva	MBN3309510.1
	Spotted gar	L_oculatus	XP_006642310.1
	European eel_1	A_anguilla_1	XP_035290913.1
	Asian arowana_1	S_formosus_1	XP_018610541.1
	Zebrafish_1	D_rerio_1	NP_001122203.1
	Northern pike_1	E_lucius_1	XP_010868725.2
	Atlantic cod_1	G_morhua_1	XP_030218275.1
	Large yellow croaker_1	L_crocea_1	XP_010748501.2
	European eel_2	A_anguilla_2	XP_035241247.1
	Asian arowana_2	S_formosus_2	XP_018598192.1
	Zebrafish_2	D_rerio_2	XP_021322033.1
	Northern pike_2	E_lucius_2	XP_010895740.1
	Atlantic cod_2	G_morhua_2	XP_030237895.1
	Large yellow croaker_2	L_crocea_2	XP_010738622.1
Orthogroup 6	Senegal bichir	P_senegalus	XP_039597875.1
	Reedfish	E_calabaricus	XP_028677513.1
	Sterlet	A_ruthenus	XP_033864467.2
	American paddlefish	P_spathula	XP_041132119.1
	Bowfin	A_calva	MBN3298773.1
	Spotted gar	L_oculatus	XP_006632440.1
	European eel_1	A_anguilla_1	XP_035288264.1
	Asian arowana_1	S_formosus_1	XP_018619586.2
	Zebrafish_1	D_rerio_1	NP_001314832.1
	Northern pike_1	E_lucius_1	XP_010896214.2
	Atlantic cod_1	G_morhua_1	XP_030212318.1
	Large yellow croaker_1	L_crocea_1	XP_019109005.2
	European eel_2	A_anguilla_2	XP_035279064.1
	Asian arowana_2	S_formosus_2	XP_029108135.1
	Zebrafish_2	D_rerio_2	XP_005170287.1
	Northern pike_2	E_lucius_2	XP_010880529.1
	Atlantic cod_2	G_morhua_2	XP_030200535.1
	Large yellow croaker_2	L_crocea_2	XP_019118791.2
Orthogroup 7	Senegal bichir	P_senegalus	XP_039620654.1
	Reedfish	E_calabaricus	XP_028671525.1
	Sterlet	A_ruthenus	XP_034766550.1
	American paddlefish	P_spathula	XP_041093798.1
	Bowfin	A_calva	MBN3309960.1
	Spotted gar	L_oculatus	XP_015215004.1
	European eel_1	A_anguilla_1	XP_035261398.1
	Asian arowana_1	S_formosus_1	XP_018615317.1
	Zebrafish_1	D_rerio_1	XP_005161865.1
	Northern pike_1	E_lucius_1	XP_028978406.1
	Atlantic cod_1	G_morhua_1	XP_030208703.1
	Large yellow croaker_1	L_crocea_1	XP_019109350.2

	European eel_2	A_anguilla_2	XP_035253025.1
	Asian arowana_2	S_formosus_2	XP_029106238.1
	Zebrafish_2	D_rerio_2	XP_009297664.1
	Northern pike_2	E_lucius_2	XP_012991360.2
	Atlantic cod_2	G_morhua_2	XP_030198122.1
	Large yellow croaker_2	L_crocea_2	XP_010734168.3
**Orthogroup 8**	Senegal bichir	P_senegalus	XP_039613429.1
	Reedfish	E_calabaricus	XP_028662234.1
	Sterlet	A_ruthenus	XP_033875661.2
	American paddlefish	P_spathula	XP_041116469.1
	Bowfin	A_calva	MBN3306223.1
	Spotted gar	L_oculatus	XP_015214519.1
	European eel_1	A_anguilla_1	XP_035248635.1
	Asian arowana_1	S_formosus_1	XP_018597171.1
	Zebrafish_1	D_rerio_1	XP_001920834.4
	Northern pike_1	E_lucius_1	XP_010889174.2
	Atlantic cod_1	G_morhua_1	XP_030199007.1
	Large yellow croaker_1	L_crocea_1	XP_019110753.2
	European eel_2	A_anguilla_2	XP_035263656.1
	Asian arowana_2	S_formosus_2	XP_018598660.1
	Zebrafish_2	D_rerio_2	NP_001154809.1
	Northern pike_2	E_lucius_2	XP_010884934.2
	Atlantic cod_2	G_morhua_2	XP_030211523.1
	Large yellow croaker_2	L_crocea_2	XP_010738541.3
**Orthogroup 9**	Senegal bichir	P_senegalus	XP_039631800.1
	Reedfish	E_calabaricus	XP_028670079.1
	Sterlet	A_ruthenus	XP_033885688.2
	American paddlefish	P_spathula	XP_041134301.1
	Bowfin	A_calva	MBN3303886.1
	Spotted gar	L_oculatus	XP_015215574.1
	European eel_1	A_anguilla_1	XP_035259521.1
	Asian arowana_1	S_formosus_1	XP_029102847.1
	Zebrafish_1	D_rerio_1	NP_001313627.1
	Northern pike_1	E_lucius_1	XP_010903886.2
	Atlantic cod_1	G_morhua_1	XP_030195443.1
	Large yellow croaker_1	L_crocea_1	XP_027144979.1
	European eel_2	A_anguilla_2	XP_035254878.1
	Asian arowana_2	S_formosus_2	XP_018610206.1
	Zebrafish_2	D_rerio_2	NP_001071052.1
	Northern pike_2	E_lucius_2	XP_010872714.3
	Atlantic cod_2	G_morhua_2	XP_030231349.1
	Large yellow croaker_2	L_crocea_2	XP_010728999.2
**Orthogroup 10**	Senegal bichir	P_senegalus	XP_039618296.1
	Reedfish	E_calabaricus	XP_028650608.1
	Sterlet	A_ruthenus	XP_033895064.2
	American paddlefish	P_spathula	XP_041125521.1
	Bowfin	A_calva	MBN3295576.1
	Spotted gar	L_oculatus	XP_006637739.1
	European eel_1	A_anguilla_1	XP_035268571.1
	Asian arowana_1	S_formosus_1	XP_018594089.1
	Zebrafish_1	D_rerio_1	NP_001002331.1
	Northern pike_1	E_lucius_1	XP_010894731.2
	Atlantic cod_1	G_morhua_1	XP_030227675.1
	Large yellow croaker_1	L_crocea_1	XP_010738094.3
	European eel_2	A_anguilla_2	XP_035276738.1
	Asian arowana_2	S_formosus_2	XP_018586276.2
	Zebrafish_2	D_rerio_2	NP_998327.1
	Northern pike_2	E_lucius_2	XP_010900099.1
	Atlantic cod_2	G_morhua_2	XP_030218923.1
	Large yellow croaker_2	L_crocea_2	XP_010741946.2

	Senegal bichir	P_senegalus	XP_039605267.1
	Reedfish	E_calabaricus	XP_028652112.1
	Sterlet	A_ruthenus	XP_033904930.1
	American paddlefish	P_spathula	XP_041084613.1
	Bowfin	A_calva	MBN3295681.1
	Spotted gar	L_oculatus	XP_015197629.1
	European eel_1	A_anguilla_1	XP_035237103.1
	Asian arowana_1	S_formosus_1	XP_018615796.1
**Orthogroup 11**	Zebrafish_1	D_rerio_1	XP_002667319.1
	Northern pike_1	E_lucius_1	XP_028980144.2
	Atlantic cod_1	G_morhua_1	XP_030220694.1
	Large yellow croaker_1	L_crocea_1	XP_027144316.1
	European eel_2	A_anguilla_2	XP_035243286.1
	Asian arowana_2	S_formosus_2	XP_018580883.1
	Zebrafish_2	D_rerio_2	XP_021332962.1
	Northern pike_2	E_lucius_2	XP_010865679.1
	Atlantic cod_2	G_morhua_2	XP_030230137.1
	Large yellow croaker_2	L_crocea_2	XP_010728409.2
	Senegal bichir	P_senegalus	XP_039598376.1
	Reedfish	E_calabaricus	XP_028675312.1
	Sterlet	A_ruthenus	XP_033895142.1
	American paddlefish	P_spathula	XP_041076027.1
	Bowfin	A_calva	MBN3300614.1
	Spotted gar	L_oculatus	XP_015212277.1
	European eel_1	A_anguilla_1	XP_035254528.1
	Asian arowana_1	S_formosus_1	XP_018618687.1
**Orthogroup 12**	Zebrafish_1	D_rerio_1	XP_017210358.1
	Northern pike_1	E_lucius_1	XP_010872279.2
	Atlantic cod_1	G_morhua_1	XP_030193039.1
	Large yellow croaker_1	L_crocea_1	XP_010728357.1
	European eel_2	A_anguilla_2	XP_035262683.1
	Asian arowana_2	S_formosus_2	XP_018600904.1
	Zebrafish_2	D_rerio_2	XP_005170178.1
	Northern pike_2	E_lucius_2	XP_010863618.2
	Atlantic cod_2	G_morhua_2	XP_030196604.1
	Large yellow croaker_2	L_crocea_2	XP_019111111.1
	Senegal bichir	P_senegalus	XP_039604495.1
	Reedfish	E_calabaricus	XP_028652928.1
	Sterlet	A_ruthenus	XP_033908125.1
	American paddlefish	P_spathula	XP_041084391.1
	Bowfin	A_calva	MBN3303092.1
	Spotted gar	L_oculatus	XP_006628635.1
	European eel_1	A_anguilla_1	XP_035238962.1
	Asian arowana_1	S_formosus_1	XP_018589389.1
**Orthogroup 13**	Zebrafish_1	D_rerio_1	XP_017212875.1
	Northern pike_1	E_lucius_1	XP_010900733.2
	Atlantic cod_1	G_morhua_1	XP_030224678.1
	Large yellow croaker_1	L_crocea_1	XP_010739068.1
	European eel_2	A_anguilla_2	XP_035244837.1
	Asian arowana_2	S_formosus_2	XP_018606233.1
	Zebrafish_2	D_rerio_2	NP_956961.2
	Northern pike_2	E_lucius_2	NP_001297780.1
	Atlantic cod_2	G_morhua_2	XP_030206298.1
	Large yellow croaker_2	L_crocea_2	XP_010751375.2
	Senegal bichir	P_senegalus	XP_039618680.1
	Reedfish	E_calabaricus	XP_028650639.1
	Sterlet	A_ruthenus	XP_034784705.1
	American paddlefish	P_spathula	XP_041120211.1
	Bowfin	A_calva	MBN3295565.1
	Spotted gar	L_oculatus	XP_015216577.1
	European eel_1	A_anguilla_1	XP_035268387.1

	Asian arowana_1	S_formosus_1	XP_018612550.2
	Zebrafish_1	D_rerio_1	XP_021327440.1
**Orthogroup 14**	Northern pike_1	E_lucius_1	XP_010901250.2
	Atlantic cod_1	G_morhua_1	XP_030228047.1
	Large yellow croaker_1	L_crocea_1	XP_019114571.2
	European eel_2	A_anguilla_2	XP_035276926.1
	Asian arowana_2	S_formosus_2	XP_029105083.1
	Zebrafish_2	D_rerio_2	XP_021322881.1
	Northern pike_2	E_lucius_2	XP_010900118.4
	Atlantic cod_2	G_morhua_2	XP_030220287.1
	Large yellow croaker_2	L_crocea_2	XP_019114571.2
	Senegal bichir	P_senegalus	XP_039618941.1
	Reedfish	E_calabaricus	XP_028649152.1
	Sterlet	A_ruthenus	XP_033894660.1
	American paddlefish	P_spathula	XP_041120504.1
	Bowfin	A_calva	MBN3295548.1
	Spotted gar	L_oculatus	XP_015216810.1
	European eel_1	A_anguilla_1	XP_035277696.1
	Asian arowana_1	S_formosus_1	XP_029104814.1
**Orthogroup 15**	Zebrafish_1	D_rerio_1	XP_017207109.1
	Northern pike_1	E_lucius_1	XP_010900121.3
	Atlantic cod_1	G_morhua_1	XP_030219244.1
	Large yellow croaker_1	L_crocea_1	XP_027147547.1
	European eel_2	A_anguilla_2	XP_035271773.1
	Asian arowana_2	S_formosus_2	XP_029106230.1
	Zebrafish_2	D_rerio_2	XP_021332809.1
	Northern pike_2	E_lucius_2	XP_034149660.1
	Atlantic cod_2	G_morhua_2	XP_030228779.1
	Large yellow croaker_2	L_crocea_2	XP_010738105.2
	Senegal bichir	P_senegalus	XP_039603762.1
	Reedfish	E_calabaricus	XP_028653499.1
	Sterlet	A_ruthenus	XP_033860962.1
	American paddlefish	P_spathula	XP_041107157.1
	Bowfin	A_calva	MBN3305733.1
	Spotted gar	L_oculatus	XP_006626327.1
	European eel_1	A_anguilla_1	XP_035280191.1
	Asian arowana_1	S_formosus_1	XP_029110285.1
**Orthogroup 16**	Zebrafish_1	D_rerio_1	NP_957090.1
	Northern pike_1	E_lucius_1	XP_010891070.1
	Atlantic cod_1	G_morhua_1	XP_030225920.1
	Large yellow croaker_1	L_crocea_1	XP_010738323.2
	European eel_2	A_anguilla_2	XP_035276759.1
	Asian arowana_2	S_formosus_2	XP_029109630.1
	Zebrafish_2	D_rerio_2	NP_956266.1
	Northern pike_2	E_lucius_2	XP_010880682.2
	Atlantic cod_2	G_morhua_2	XP_030201824.1
	Large yellow croaker_2	L_crocea_2	XP_010737603.1
	Reedfish	E_calabaricus	XP_028653826.1
	Sterlet	A_ruthenus	/
	American paddlefish	P_spathula	XP_041094114.1
	Bowfin	A_calva	MBN3298311.1
	Spotted gar	L_oculatus	XP_006627569.1
	European eel_1	A_anguilla_1	XP_035264968.1
	Asian arowana_1	S_formosus_1	XP_018588898.1
	Zebrafish_1	D_rerio_1	XP_021333479.1
	Northern pike_1	E_lucius_1	XP_010898119.1
	Atlantic cod_1	G_morhua_1	XP_030194559.1
	Large yellow croaker_1	L_crocea_1	XP_010751620.2
	European eel_2	A_anguilla_2	XP_035289078.1
	Asian arowana_2	S_formosus_2	XP_018607236.2
	Zebrafish_2	D_rerio_2	XP_009303554.1

	Northern pike_2	E_lucius_2	XP_034149175.1
	Atlantic cod_2	G_morhua_2	XP_030217658.1
	Large yellow croaker_2	L_crocea_2	XP_019122538.1
Orthogroup 18	Senegal bichir	P_senegalus	XP_039601806.1
	Reedfish	E_calabaricus	XP_028655066.1
	Sterlet	A_ruthenus	XP_033864658.1
	American paddlefish	P_spathula	XP_041128678.1
	Bowfin	A_calva	MBN3306179.1
	Spotted gar	L_oculatus	XP_006628329.1
	European eel_1	A_anguilla_1	XP_035269062.1
	Asian arowana_1	S_formosus_1	XP_018616530.1
	Zebrafish_1	D_rerio_1	XP_001342691.1
	Northern pike_1	E_lucius_1	XP_010888390.1
	Atlantic cod_1	G_morhua_1	XP_030204780.1
	Large yellow croaker_1	L_crocea_1	XP_027130150.1
	European eel_2	A_anguilla_2	XP_035287649.1
	Asian arowana_2	S_formosus_2	XP_018590246.1
	Zebrafish_2	D_rerio_2	NP_001003499.1
	Northern pike_2	E_lucius_2	XP_010898189.1
	Atlantic cod_2	G_morhua_2	XP_030217965.1
	Large yellow croaker_2	L_crocea_2	XP_010736283.2
Orthogroup 19	Senegal bichir	P_senegalus	XP_039630487.1
	Reedfish	E_calabaricus	XP_028670134.1
	Sterlet	A_ruthenus	XP_034782997.1
	American paddlefish	P_spathula	XP_041133772.1
	Bowfin	A_calva	MBN3305094.1
	Spotted gar	L_oculatus	XP_015215342.1
	European eel_1	A_anguilla_1	XP_035254641.1
	Asian arowana_1	S_formosus_1	XP_029106280.1
	Zebrafish_1	D_rerio_1	XP_021324946.1
	Northern pike_1	E_lucius_1	XP_010887736.1
	Atlantic cod_1	G_morhua_1	XP_030203983.1
	Large yellow croaker_1	L_crocea_1	XP_019113590.1
	European eel_2	A_anguilla_2	XP_035259243.1
	Asian arowana_2	S_formosus_2	XP_018600885.2
	Zebrafish_2	D_rerio_2	XP_009294691.1
	Northern pike_2	E_lucius_2	XP_028978189.2
	Atlantic cod_2	G_morhua_2	XP_030208000.1
	Large yellow croaker_2	L_crocea_2	XP_027139429.1
Orthogroup 20	Senegal bichir	P_senegalus	XP_039623482.1
	Reedfish	E_calabaricus	XP_028666477.1
	Sterlet	A_ruthenus	/
	American paddlefish	P_spathula	XP_041088055.1
	Bowfin	A_calva	MBN3305410.1
	Spotted gar	L_oculatus	XP_006634229.1
	European eel_1	A_anguilla_1	XP_035281662.1
	Asian arowana_1	S_formosus_1	XP_018610445.2
	Zebrafish_1	D_rerio_1	NP_571698.1
	Northern pike_1	E_lucius_1	XP_010900255.2
	Atlantic cod_1	G_morhua_1	XP_030225038.1
	Large yellow croaker_1	L_crocea_1	XP_019129955.2
	European eel_2	A_anguilla_2	XP_035273078.1
	Asian arowana_2	S_formosus_2	XP_018593716.1
	Zebrafish_2	D_rerio_2	NP_945337.3
	Northern pike_2	E_lucius_2	XP_010889922.2
	Atlantic cod_2	G_morhua_2	XP_030208527.1
	Large yellow croaker_2	L_crocea_2	XP_027138404.1
	Senegal bichir	P_senegalus	XP_039632493.1
	Reedfish	E_calabaricus	XP_028648575.1
	Sterlet	A_ruthenus	XP_034781931.1

	American paddlefish	P_spathula	XP_041116588.1
	Bowfin	A_calva	MBN3302748.1
	Spotted gar	L_oculatus	XP_015201714.1
	European eel_1	A_anguilla_1	XP_035259557.1
	Asian arowana_1	S_formosus_1	XP_018616533.2
**Orthogroup 21**	Zebrafish_1	D_rerio_1	NP_001036218.1
	Northern pike_1	E_lucius_1	XP_010867627.1
	Atlantic cod_1	G_morhua_1	XP_030196236.1
	Large yellow croaker_1	L_crocea_1	XP_010739778.2
	European eel_2	A_anguilla_2	XP_035255326.1
	Asian arowana_2	S_formosus_2	XP_018593552.1
	Zebrafish_2	D_rerio_2	NP_001018174.2
	Northern pike_2	E_lucius_2	XP_010902252.2
	Atlantic cod_2	G_morhua_2	XP_030235209.1
	Large yellow croaker_2	L_crocea_2	XP_010743516.3
	Senegal bichir	P_senegalus	XP_039622095.1
	Reedfish	E_calabaricus	XP_028671641.1
	Sterlet	A_ruthenus	XP_033876376.2
	American paddlefish	P_spathula	XP_041109909.1
	Bowfin	A_calva	MBN3297464.1
	Spotted gar	L_oculatus	XP_015207009.1
	European eel_1	A_anguilla_1	XP_035291073.1
	Asian arowana_1	S_formosus_1	XP_018598785.1
**Orthogroup 22**	Zebrafish_1	D_rerio_1	NP_001035400.1
	Northern pike_1	E_lucius_1	XP_010893534.1
	Atlantic cod_1	G_morhua_1	XP_030216625.1
	Large yellow croaker_1	L_crocea_1	XP_010746705.1
	European eel_2	A_anguilla_2	XP_035267505.1
	Asian arowana_2	S_formosus_2	XP_029112997.1
	Zebrafish_2	D_rerio_2	NP_878288.2
	Northern pike_2	E_lucius_2	XP_010901464.1
	Atlantic cod_2	G_morhua_2	XP_030223472.1
	Large yellow croaker_2	L_crocea_2	XP_010734903.3
	Senegal bichir	P_senegalus	XP_039609323.1
	Reedfish	E_calabaricus	XP_028659803.1
	Sterlet	A_ruthenus	XP_033855044.2
	American paddlefish	P_spathula	XP_041101294.1
	Bowfin	A_calva	MBN3298967.1
	Spotted gar	L_oculatus	XP_006634631.1
	European eel_1	A_anguilla_1	XP_035270875.1
	Asian arowana_1	S_formosus_1	XP_018615810.2
**Orthogroup 23**	Zebrafish_1	D_rerio_1	NP_775390.2
	Northern pike_1	E_lucius_1	XP_010870745.1
	Atlantic cod_1	G_morhua_1	XP_030229202.1
	Large yellow croaker_1	L_crocea_1	XP_010740165.1
	European eel_2	A_anguilla_2	XP_035287429.1
	Asian arowana_2	S_formosus_2	XP_018613131.2
	Zebrafish_2	D_rerio_2	NP_001091727.1
	Northern pike_2	E_lucius_2	XP_010882597.1
	Atlantic cod_2	G_morhua_2	XP_030220660.1
	Large yellow croaker_2	L_crocea_2	XP_010742124.3
	Senegal bichir	P_senegalus	XP_039597759.1
	Reedfish	E_calabaricus	XP_028677504.1
	Sterlet	A_ruthenus	XP_034761093.1
	American paddlefish	P_spathula	XP_041121848.1
	Bowfin	A_calva	MBN3305050.1
	Spotted gar	L_oculatus	XP_006632604.1
	European eel_1	A_anguilla_1	XP_035281302.1
	Asian arowana_1	S_formosus_1	XP_018583359.1
**Orthogroup 24**	Zebrafish_1	D_rerio_1	XP_009291625.1
	Northern pike_1	E_lucius_1	XP_010877173.1

	Atlantic cod_1	G_morhua_1	XP_030212580.1
	Large yellow croaker_1	L_crocea_1	XP_010732218.3
	European eel_2	A_anguilla_2	XP_035276210.1
	Asian arowana_2	S_formosus_2	XP_018580653.2
	Zebrafish_2	D_rerio_2	NP_001038536.2
	Northern pike_2	E_lucius_2	XP_010880747.1
	Atlantic cod_2	G_morhua_2	XP_030201548.1
	Large yellow croaker_2	L_crocea_2	XP_027132003.1
	Senegal bichir	P_senegalus	XP_039623369.1
	Reedfish	E_calabaricus	XP_028650936.1
	Sterlet	A_ruthenus	XP_034773571.1
	American paddlefish	P_spathula	XP_041094884.1
	Bowfin	A_calva	MBN3304378.1
	Spotted gar	L_oculatus	/
	European eel_1	A_anguilla_1	XP_035281142.1
	Asian arowana_1	S_formosus_1	XP_018592537.1
**Orthogroup 25**	Zebrafish_1	D_rerio_1	XP_693668.1
	Northern pike_1	E_lucius_1	XP_010883114.1
	Atlantic cod_1	G_morhua_1	XP_030226405.1
	Large yellow croaker_1	L_crocea_1	XP_027142774.1
	European eel_2	A_anguilla_2	XP_035285477.1
	Asian arowana_2	S_formosus_2	XP_018603280.1
	Zebrafish_2	D_rerio_2	NP_705957.1
	Northern pike_2	E_lucius_2	XP_010863806.1
	Atlantic cod_2	G_morhua_2	XP_030202656.1
	Large yellow croaker_2	L_crocea_2	XP_010741811.1
	Senegal bichir	P_senegalus	XP_039593564.1
	Reedfish	E_calabaricus	XP_028673697.1
	Sterlet	A_ruthenus	XP_033855356.1
	American paddlefish	P_spathula	XP_041100742.1
	Bowfin	A_calva	MBN3305527.1
	Spotted gar	L_oculatus	XP_006635602.1
	European eel_1	A_anguilla_1	XP_035276386.1
	Asian arowana_1	S_formosus_1	XP_018610201.1
**Orthogroup 26**	Zebrafish_1	D_rerio_1	XP_021322479.1
	Northern pike_1	E_lucius_1	XP_010864926.1
	Atlantic cod_1	G_morhua_1	XP_030215390.1
	Large yellow croaker_1	L_crocea_1	XP_010752540.1
	European eel_2	A_anguilla_2	XP_035286694.1
	Asian arowana_2	S_formosus_2	XP_018621413.1
	Zebrafish_2	D_rerio_2	XP_003200569.1
	Northern pike_2	E_lucius_2	XP_010898749.2
	Atlantic cod_2	G_morhua_2	XP_030203331.1
	Large yellow croaker_2	L_crocea_2	XP_010733680.2
	Senegal bichir	P_senegalus	XP_039600844.1
	Reedfish	E_calabaricus	XP_028646314.1
	Sterlet	A_ruthenus	XP_033868149.2
	American paddlefish	P_spathula	XP_041128032.1
	Bowfin	A_calva	MBN3302909.1
	Spotted gar	L_oculatus	XP_006628312.1
	European eel_1	A_anguilla_1	XP_035241675.1
	Asian arowana_1	S_formosus_1	XP_018619656.2
**Orthogroup 27**	Zebrafish_1	D_rerio_1	XP_009290217.1
	Northern pike_1	E_lucius_1	XP_010872263.2
	Atlantic cod_1	G_morhua_1	XP_030236171.1
	Large yellow croaker_1	L_crocea_1	XP_027135877.1
	European eel_2	A_anguilla_2	XP_035289285.1
	Asian arowana_2	S_formosus_2	XP_029106896.1
	Zebrafish_2	D_rerio_2	XP_001924044.3
	Northern pike_2	E_lucius_2	XP_010895542.2
	Atlantic cod_2	G_morhua_2	XP_030218174.1

	Large yellow croaker_2	L_crocea_2	XP_027129271.1
**Orthogroup 28**	Senegal bichir	P_senegalus	XP_039614704.1
	Reedfish	E_calabaricus	XP_028650417.1
	Sterlet	A_ruthenus	XP_033884405.2
	American paddlefish	P_spathula	XP_041120412.1
	Bowfin	A_calva	MBN3297103.1
	Spotted gar	L_oculatus	XP_015216319.1
	European eel_1	A_anguilla_1	XP_035269009.1
	Asian arowana_1	S_formosus_1	XP_018612655.1
	Zebrafish_1	D_rerio_1	XP_005172907.1
	Northern pike_1	E_lucius_1	XP_010895232.1
	Atlantic cod_1	G_morhua_1	XP_019898016.2
	Large yellow croaker_1	L_crocea_1	XP_027145714.1
	European eel_2	A_anguilla_2	XP_035276628.1
	Asian arowana_2	S_formosus_2	XP_029105181.1
	Zebrafish_2	D_rerio_2	NP_001013543.1
	Northern pike_2	E_lucius_2	XP_019898016.2
	Atlantic cod_2	G_morhua_2	XP_030219432.1
	Large yellow croaker_2	L_crocea_2	XP_027141144.1
**Orthogroup 29**	Senegal bichir	P_senegalus	XP_039609246.1
	Reedfish	E_calabaricus	XP_028659109.1
	Sterlet	A_ruthenus	XP_033848480.1
	American paddlefish	P_spathula	XP_041103234.1
	Bowfin	A_calva	MBN3305013.1
	Spotted gar	L_oculatus	XP_015210378.1
	European eel_1	A_anguilla_1	XP_035271322.1
	Asian arowana_1	S_formosus_1	XP_018609729.1
	Zebrafish_1	D_rerio_1	NP_001268920.1
	Northern pike_1	E_lucius_1	XP_010888460.2
	Atlantic cod_1	G_morhua_1	XP_030204819.1
	Large yellow croaker_1	L_crocea_1	XP_027129995.1
	European eel_2	A_anguilla_2	XP_035285859.1
	Asian arowana_2	S_formosus_2	XP_018583401.2
	Zebrafish_2	D_rerio_2	NP_001289171.1
	Northern pike_2	E_lucius_2	XP_010892379.1
	Atlantic cod_2	G_morhua_2	XP_030219392.1
	Large yellow croaker_2	L_crocea_2	XP_010752670.2
**Orthogroup 30**	Senegal bichir	P_senegalus	XP_039616843.1
	Reedfish	E_calabaricus	XP_028666776.1
	Sterlet	A_ruthenus	XP_033881524.2
	American paddlefish	P_spathula	XP_041110400.1
	Bowfin	A_calva	MBN3298345.1
	Spotted gar	L_oculatus	XP_015194475.1
	European eel_1	A_anguilla_1	XP_035283275.1
	Asian arowana_1	S_formosus_1	XP_029107956.1
	Zebrafish_1	D_rerio_1	NP_851300.2
	Northern pike_1	E_lucius_1	XP_010881894.1
	Atlantic cod_1	G_morhua_1	XP_030221904.1
	Large yellow croaker_1	L_crocea_1	XP_019128196.1
	European eel_2	A_anguilla_2	XP_035258166.1
	Asian arowana_2	S_formosus_2	XP_018603052.1
	Zebrafish_2	D_rerio_2	NP_001014814.1
	Northern pike_2	E_lucius_2	XP_012994152.2
	Atlantic cod_2	G_morhua_2	XP_030198083.1
	Large yellow croaker_2	L_crocea_2	XP_019116039.1
