# Supplementary material for: Molecular Dating of the Teleost Whole Genome Duplication (3R) Is Compatible With the Expectations of Delayed Rediploidization
Source: Genome Biol Evol. 2024 Jun 24;16(7):evae128. doi: 10.1093/gbe/evae128 (PMC11259977; doi:10.1093/gbe/evae128)
Supplement: evae128_Supplementary_Data [file evae128_supplementary_data.zip › 4_Supplementary_material_GBE.docx]

**Supplementary material**

1. **Supplementary tables**

**1.1 Supplementary Table 1**. Age estimates of different nodes from our largest dataset (with 30 orthogroups concatenated) with ILN (independent log normal/independent rates) clock **(a)** and GBM (geometric Brownian motion/autocorrelated rates) clock **(b)**.

**(a) ILN clock**

| **Name** | | **Description** | **Estimates from ILN clock** | | | |
| --- | --- | --- | --- | --- | --- | --- |
|  |  |  | ILN_mean (100 Mya) | 95% HPD (100 Mya) | | Interval (100 Myr) |
|  |  |  |  | Maximum | Minimum |  |
| Node 1 | | Crown Actinopterygii | 3.7801 | 3.9170 | 3.6722 | 0.2448 |
| Node 2 | | Crown Cladistia | 0.1646 | 0.2037 | 0.1294 | 0.0743 |
| Node 3 | | Crown Actinopteri | 3.718 | 3.7737 | 3.6546 | 0.1191 |
| Node 4 | | Crown Chondrostei | 1.2187 | 1.2402 | 1.208 | 0.0322 |
| Node 5 | | Crown Neopterygii | 3.2203 | 3.3133 | 3.1276 | 0.1857 |
| Node 6 | | Crown Holostei | 2.8761 | 3.0382 | 2.7133 | 0.3249 |
| Node 7 | | Ts3R | 2.7682 | 2.8618 | 2.672 | 0.1898 |
| **Ts3R Orthogroup 1** | Node 8 | Crown Teleostei | 2.4455 | 2.5436 | 2.3416 | 0.202 |
|  | Node 9 | Divergence of Osteoglossomorpha and Elopocephalai | 2.2565 | 2.3788 | 2.1305 | 0.2483 |
|  | Node 10 | Crown Clupeocephala | 1.9734 | 2.088 | 1.8601 | 0.2279 |
|  | Node 11 | Divergence of Protacanthopterygii and (Neoteleostei+Stomiati) | 1.604 | 1.7041 | 1.5094 | 0.1947 |
|  | Node 12 | Crown Acanthomorpha | 1.1533 | 1.2796 | 1.0351 | 0.2445 |
| **Ts3R Orthogroup 2** | Node 13 | Crown Teleostei | 2.4525 | 2.5418 | 2.3529 | 0.1889 |
|  | Node 14 | Divergence of Osteoglossomorpha and Elopocephalai | 2.2743 | 2.3912 | 2.1499 | 0.2413 |
|  | Node 15 | Crown Clupeocephala | 2.0041 | 2.1083 | 1.9024 | 0.2059 |
|  | Node 16 | Divergence of Protacanthopterygii and (Neoteleostei+Stomiati) | 1.585 | 1.6749 | 1.5094 | 0.1655 |
|  | Node 17 | Crown Acanthomorpha | 1.1387 | 1.2541 | 1.0289 | 0.2252 |

**(b) GBM clock**

| **Name** | | **Description** | **Estimates from GBM clock** | | | |
| --- | --- | --- | --- | --- | --- | --- |
|  |  |  | GBM_mean (100 Mya) | 95% HPD (100 Mya) | | Interval (100 Myr) |
|  |  |  |  | Maximum | Minimum |  |
| Node 1 | | Crown Actinopterygii | 3.8291 | 4.0098 | 3.7052 | 0.3046 |
| Node 2 | | Crown Cladistia | 0.392 | 0.5173 | 0.275 | 0.2423 |
| Node 3 | | Crown Actinopteri | 3.7428 | 3.8109 | 3.6899 | 0.121 |
| Node 4 | | Crown Chondrostei | 1.2495 | 1.3261 | 1.208 | 0.1181 |
| Node 5 | | Crown Neopterygii | 3.1555 | 3.2332 | 3.084 | 0.1492 |
| Node 6 | | Crown Holostei | 2.8987 | 2.999 | 2.7946 | 0.2044 |
| Node 7 | | Ts3R | 2.5832 | 2.6545 | 2.5168 | 0.1377 |
| **Ts3R Orthogroup 1** | Node 8 | Crown Teleostei | 2.3179 | 2.3833 | 2.251 | 0.1323 |
|  | Node 9 | Divergence of Osteoglossomorpha and Elopocephalai | 2.1763 | 2.2504 | 2.1011 | 0.1493 |
|  | Node 10 | Crown Clupeocephala | 1.8683 | 1.9283 | 1.8133 | 0.115 |
|  | Node 11 | Divergence of Protacanthopterygii and (Neoteleostei+Stomiati) | 1.532 | 1.5717 | 1.5094 | 0.0623 |
|  | Node 12 | Crown Acanthomorpha | 1.1204 | 1.1833 | 1.0579 | 0.1254 |
| **Ts3R Orthogroup 2** | Node 13 | Crown Teleostei | 2.283 | 2.3468 | 2.221 | 0.1258 |
|  | Node 14 | Divergence of Osteoglossomorpha and Elopocephalai | 2.1773 | 2.2462 | 2.1071 | 0.1391 |
|  | Node 15 | Crown Clupeocephala | 1.8658 | 1.9131 | 1.8181 | 0.095 |
|  | Node 16 | Divergence of Protacanthopterygii and (Neoteleostei+Stomiati) | 1.5196 | 1.54 | 1.5094 | 0.0306 |
|  | Node 17 | Crown Acanthomorpha | 1.1168 | 1.1742 | 1.0606 | 0.1136 |

**1.2 Supplementary Table 2.** Age estimate of 3R event based on different datasets (individual orthogroups and datasets concatenating different numbers of orthogroups) with ILN (independent log normal/independent rates) clock.

| **Dataset** | **Estimates with ILN clock (100 Mya)** | | | |
| --- | --- | --- | --- | --- |
|  | **Mean** | **95% HPD** | | |
|  |  | **Maximum** | **Minimum** | **Interval (100 Myr)** |
| Orthogroup 1 | 2.7181 | 3.0002 | 2.457 | 0.5432 |
| Orthogroup 2 | 2.8833 | 3.1681 | 2.6045 | 0.5636 |
| Orthogroup 3 | 2.6527 | 2.8857 | 2.4252 | 0.4605 |
| Orthogroup 4 | 2.556 | 2.7946 | 2.32 | 0.4746 |
| Orthogroup 5 | 2.6765 | 2.9274 | 2.445 | 0.4824 |
| Orthogroup 6 | 2.7403 | 3.0284 | 2.4706 | 0.5578 |
| Orthogroup 7 | 2.6502 | 2.9198 | 2.421 | 0.4988 |
| Orthogroup 8 | 2.6842 | 2.9314 | 2.4419 | 0.4895 |
| Orthogroup 9 | 2.6508 | 2.877 | 2.443 | 0.434 |
| Orthogroup 10 | 2.7667 | 3.0395 | 2.4842 | 0.5553 |
| Orthogroup 11 | 2.7966 | 3.0468 | 2.5282 | 0.5186 |
| Orthogroup 12 | 2.766 | 3.0657 | 2.4863 | 0.5794 |
| Orthogroup 13 | 2.6533 | 2.9275 | 2.4082 | 0.5193 |
| Orthogroup 14 | 2.77 | 3.0605 | 2.4953 | 0.5652 |
| Orthogroup 15 | 2.7688 | 3.0604 | 2.4889 | 0.5715 |
| Orthogroup 16 | 2.7654 | 3.0477 | 2.4935 | 0.5542 |
| Orthogroup 17 | 2.7403 | 3.035 | 2.4791 | 0.5559 |
| Orthogroup 18 | 2.7189 | 2.9892 | 2.4614 | 0.5278 |
| Orthogroup 19 | 2.7108 | 3.001 | 2.4457 | 0.5553 |
| Orthogroup 20 | 2.7643 | 3.0746 | 2.4839 | 0.5907 |
| Orthogroup 21 | 2.7031 | 2.9635 | 2.4589 | 0.5046 |
| Orthogroup 22 | 2.6571 | 2.9075 | 2.4011 | 0.5064 |
| Orthogroup 23 | 2.7467 | 3.0327 | 2.4827 | 0.55 |
| Orthogroup 24 | 2.81 | 3.1043 | 2.5327 | 0.5716 |
| Orthogroup 25 | 2.6656 | 2.9227 | 2.4116 | 0.5111 |
| Orthogroup 26 | 2.6138 | 2.9063 | 2.3652 | 0.5411 |
| Orthogroup 27 | 2.6746 | 2.9261 | 2.4354 | 0.4907 |
| Orthogroup 28 | 2.6729 | 2.9353 | 2.4153 | 0.52 |
| Orthogroup 29 | 2.6079 | 2.8187 | 2.4049 | 0.4138 |
| Orthogroup 30 | 2.7511 | 3.0189 | 2.5047 | 0.5142 |
| Orthogroup 1-6 concatenated | 2.7272 | 2.886 | 2.5623 | 0.3237 |
| Orthogroup 1-12 concatenated | 2.7513 | 2.8771 | 2.6201 | 0.257 |
| Orthogroup 1-18 concatenated | 2.7607 | 2.8755 | 2.6422 | 0.2333 |
| Orthogroup 1-30 concatenated | 2.7682 | 2.8618 | 2.672 | 0.1898 |

**1.3 Supplementary Table 3.** Gene ontology terms enriched in our dataset. (a) Biological process gene; (b) Cellular component; (c) Molecular function ontology enriched for the 60 zebrafish ohnologue sequences from our 30 ohnologue pairs datasets.

1. Biological process

| **GO ID** | **KEGG pathway** | **Enrichment** | **p-value** | **FDR** | **Genes** | **Ohnolog** | **Ancestral chromosome** |
| --- | --- | --- | --- | --- | --- | --- | --- |
| GO:0030148 | sphingolipid biosynthetic process | 59.396 | 5.0826e-7 | 0.0020208 | cers2a | Ohnolog 25 | 2a |
|  |  |  |  |  | cers2b |  | 2b |
|  |  |  |  |  | elovl4a | Ohnolog 20 | 2b |
|  |  |  |  |  | elovl4b |  | 1a |

1. Cellular component

| **GO ID** | **KEGG pathway** | **Enrichment** | **p-value** | **FDR** | **Genes** | **Ohnolog** | **Ancestral chromosome** |
| --- | --- | --- | --- | --- | --- | --- | --- |
| GO:0005604 | basement membrane | 130.69 | 9.5185×10^-5^ | 0.052779 | loxl2a | Ohnolog 2 | 9b |
|  |  |  |  |  | loxl2b |  | 9a |

1. Molecular function

| **GO ID** | **KEGG pathway** | **Enrichment** | **p-value** | **FDR** | **Genes** | **Ohnolog** | **Ancestral chromosome** |
| --- | --- | --- | --- | --- | --- | --- | --- |
| GO:0004994 | somatostatin receptor activity | 167.95 | 5.4128×10^-5^ | 0.018376 | sstr2a | Ohnolog 12 | 5b |
|  |  |  |  |  | sstr2b |  | 5a |

**1.4 Supplementary Table 4.** KEGG pathway enriched in our dataset.

| **KEGG ID** | **KEGG pathway** | **Enrichment** | **p-value** | **FDR** | **Genes** | **Ohnolog** | **Ancestral chromosome** |
| --- | --- | --- | --- | --- | --- | --- | --- |
| dre04080 | Neuroactive ligand-receptor interaction | 5.2398 | 5.589×10^-4^ | 0.089983 | chrna9 | Ohnolog 3 | 6a |
|  |  |  |  |  | LOC798522 |  | 6b |
|  |  |  |  |  | p2rx3a | Ohnolog 20 | 6b |
|  |  |  |  |  | p2rx3b |  | 6a |
|  |  |  |  |  | sstr2a | Ohnolog 12 | 5b |
|  |  |  |  |  | sstr2b |  | 5a |

**1.5 Supplementary Table 5. Please refer to the separate file for the gene IDs corresponding to each sequence in our dataset.**

**1.6 Supplementary Table 6. Please refer to the separate file for detailed information (IDs, functions, locations and synteny) of each ohnolog.**

**1.7 Supplementary Table 7**. Information of the genomes used in this study.

| **Taxon** | **Species** | **Assembly name** | **Accession number** |
| --- | --- | --- | --- |
| Cladistia | *Polypterus senegalus* | ASM1683550v1 | GCF_016835505.1 |
|  | *Erpetoichthys calabaricus* | fErpCal1.1 | GCF_900747795.1 |
| Chondrostei | *Acipenser ruthenus* | ASM1064508v1 | GCF_010645085.1 |
|  | *Polyodon spathula* | ASM1765450v1 | GCF_017654505.1 |
| Holostei | *Lepisosteus oculatus* | LepOcu1 | GCF_000242695.1 |
|  | *Amia calva* | BGI_Acal_1.1 | GCA_016984155.1 |
| Teleostei | *Scleropages formosus* | fSclFor1.1 | GCF_900964775.1 |
|  | *Anguilla anguilla* | fAngAng1.pri | GCF_013347855.1 |
|  | *Danio rerio* | GRCz11 | GCF_000002035.6 |
|  | *Esox lucius* | fEsoLuc1.pri | GCF_011004845.1 |
|  | *Gadus morhua* | gadMor3.0 | GCF_902167405.1 |
|  | *Larimichthys crocea* | L_crocea_2.0 | GCF_000972845.2 |

**1.8 Supplementary Table 8.** Basic information of alignments of individual orthogroups

| **Orthogroups** | **Number of sequences** | **Alignment length (aa)** |
| --- | --- | --- |
| Orthogroup 1 | 18 | 453 |
| Orthogroup 2 | 18 | 738 |
| Orthogroup 3 | 18 | 477 |
| Orthogroup 4 | 18 | 350 |
| Orthogroup 5 | 18 | 439 |
| Orthogroup 6 | 18 | 234 |
| Orthogroup 7 | 18 | 311 |
| Orthogroup 8 | 18 | 739 |
| Orthogroup 9 | 18 | 480 |
| Orthogroup 10 | 18 | 427 |
| Orthogroup 11 | 18 | 569 |
| Orthogroup 12 | 18 | 372 |
| Orthogroup 13 | 18 | 233 |
| Orthogroup 14 | 18 | 2117 |
| Orthogroup 15 | 18 | 1025 |
| Orthogroup 16 | 18 | 302 |
| Orthogroup 17 | 17 | 366 |
| Orthogroup 18 | 18 | 448 |
| Orthogroup 19 | 18 | 401 |
| Orthogroup 20 | 17 | 390 |
| Orthogroup 21 | 18 | 646 |
| Orthogroup 22 | 18 | 498 |
| Orthogroup 23 | 18 | 238 |
| Orthogroup 24 | 18 | 398 |
| Orthogroup 25 | 17 | 378 |
| Orthogroup 26 | 18 | 195 |
| Orthogroup 27 | 18 | 1533 |
| Orthogroup 28 | 18 | 227 |
| Orthogroup 29 | 18 | 1310 |
| Orthogroup 30 | 18 | 992 |
| Orthogroup 1-6 concatenated | 108 | 2691 |
| Orthogroup 1-12 concatenated | 216 | 5589 |
| Orthogroup 1-18 concatenated | 323 | 10080 |
| Orthogroup 1-30 concatenated | 537 | 17286 |

- 1. **Supplementary Table 9.** Amino acid substitution model and alpha used for different orthogroups in codeml.

| **Orthogroups** | **aa substitution model used in codeml** | **Alpha** |
| --- | --- | --- |
| Orthogroup 1 | JTT+G4 | 0.481 |
| Orthogroup 2 | JTTDCMut+G4 | 0.43 |
| Orthogroup 3 | JTT+G4 | 0.281 |
| Orthogroup 4 | JTT+G4 | 0.746 |
| Orthogroup 5 | JTT+G4 | 0.824 |
| Orthogroup 6 | JTT+G4 | 1.161 |
| Orthogroup 7 | JTT+G4 | 1.07 |
| Orthogroup 8 | LG+G4 | 0.646 |
| Orthogroup 9 | JTT+G4 | 1.117 |
| Orthogroup 10 | JTTDCMut+G4 | 0.575 |
| Orthogroup 11 | JTT+G4 | 0.395 |
| Orthogroup 12 | JTT+G4 | 0.46 |
| Orthogroup 13 | JTTDCMut+G4 | 0.565 |
| Orthogroup 14 | JTT+G4 | 0.833 |
| Orthogroup 15 | JTT+G4 | 0.732 |
| Orthogroup 16 | LG+G4 | 0.357 |
| Orthogroup 17 | JTT+G4 | 0.443 |
| Orthogroup 18 | LG+G4 | 0.84 |
| Orthogroup 19 | JTT+G4 | 0.599 |
| Orthogroup 20 | LG+G4 | 0.514 |
| Orthogroup 21 | JTT+G4 | 0.507 |
| Orthogroup 22 | JTTDCMut+G4 | 0.693 |
| Orthogroup 23 | JTTDCMut+G4 | 1.21 |
| Orthogroup 24 | LG+G4 | 1.233 |
| Orthogroup 25 | LG+G4 | 0.691 |
| Orthogroup 26 | JTT+G4 | 0.978 |
| Orthogroup 27 | JTT+G4 | 0.531 |
| Orthogroup 28 | JTT+G4 | 1.011 |
| Orthogroup 29 | JTT+G4 | 0.613 |
| Orthogroup 30 | JTTDCMut+G4 | 0.973 |

- 1. **Supplementary Table 10.** The alpha and beta parameters of the gamma distribution used in modeling the evolutionary rate difference across orthogroups.

| **Orthogroups** | **Gamma_Alpha** | **Gamma_Beta** |
| --- | --- | --- |
| Orthogroup 1 | 2 | 17.49 |
| Orthogroup 2 | 2 | 13.14 |
| Orthogroup 3 | 2 | 9.06 |
| Orthogroup 4 | 2 | 4.54 |
| Orthogroup 5 | 2 | 5.66 |
| Orthogroup 6 | 2 | 6.32 |
| Orthogroup 7 | 2 | 9.11 |
| Orthogroup 8 | 2 | 7.26 |
| Orthogroup 9 | 2 | 4.28 |
| Orthogroup 10 | 2 | 10.26 |
| Orthogroup 11 | 2 | 10.21 |
| Orthogroup 12 | 2 | 9.35 |
| Orthogroup 13 | 2 | 7.14 |
| Orthogroup 14 | 2 | 7.17 |
| Orthogroup 15 | 2 | 4.74 |
| Orthogroup 16 | 2 | 15.86 |
| Orthogroup 17 | 2 | 7.25 |
| Orthogroup 18 | 2 | 5.77 |
| Orthogroup 19 | 2 | 9.09 |
| Orthogroup 20 | 2 | 6.87 |
| Orthogroup 21 | 2 | 12.3 |
| Orthogroup 22 | 2 | 4.61 |
| Orthogroup 23 | 2 | 6.51 |
| Orthogroup 24 | 2 | 4.05 |
| Orthogroup 25 | 2 | 12.33 |
| Orthogroup 26 | 2 | 6.65 |
| Orthogroup 27 | 2 | 8.74 |
| Orthogroup 28 | 2 | 8.42 |
| Orthogroup 29 | 2 | 12.78 |
| Orthogroup 30 | 2 | 7.87 |

**2. Supplementary Figures**

**2.1 Supplementary Figure 1**

**
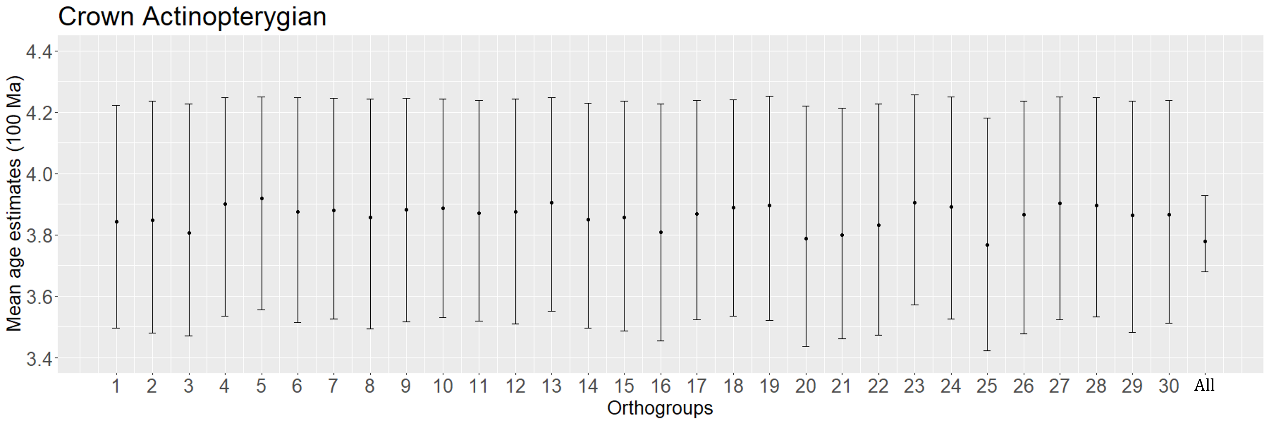

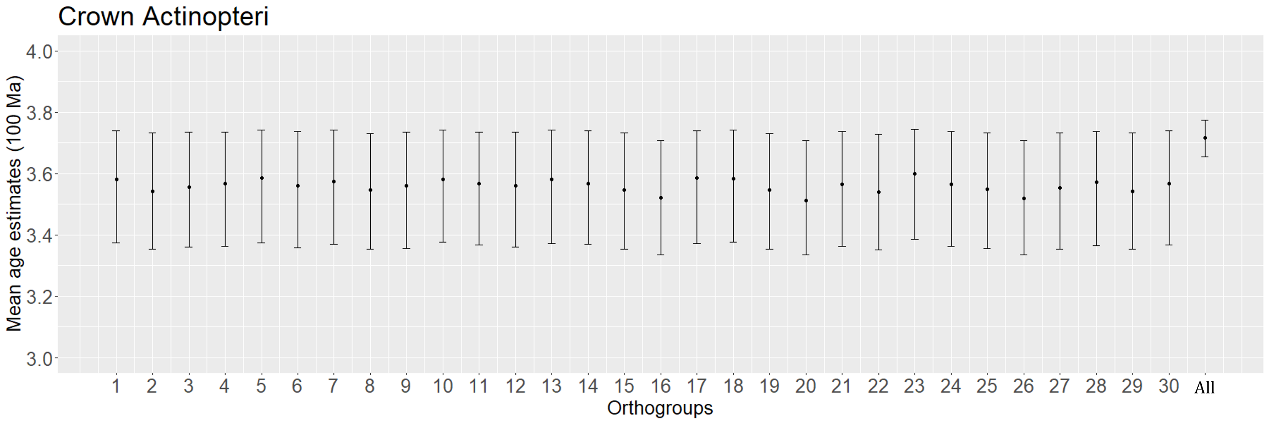

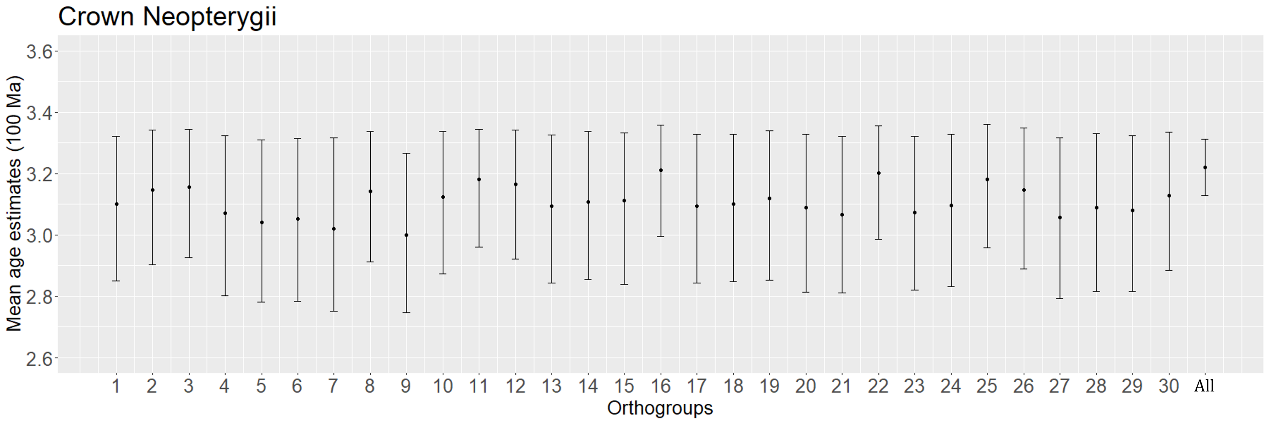

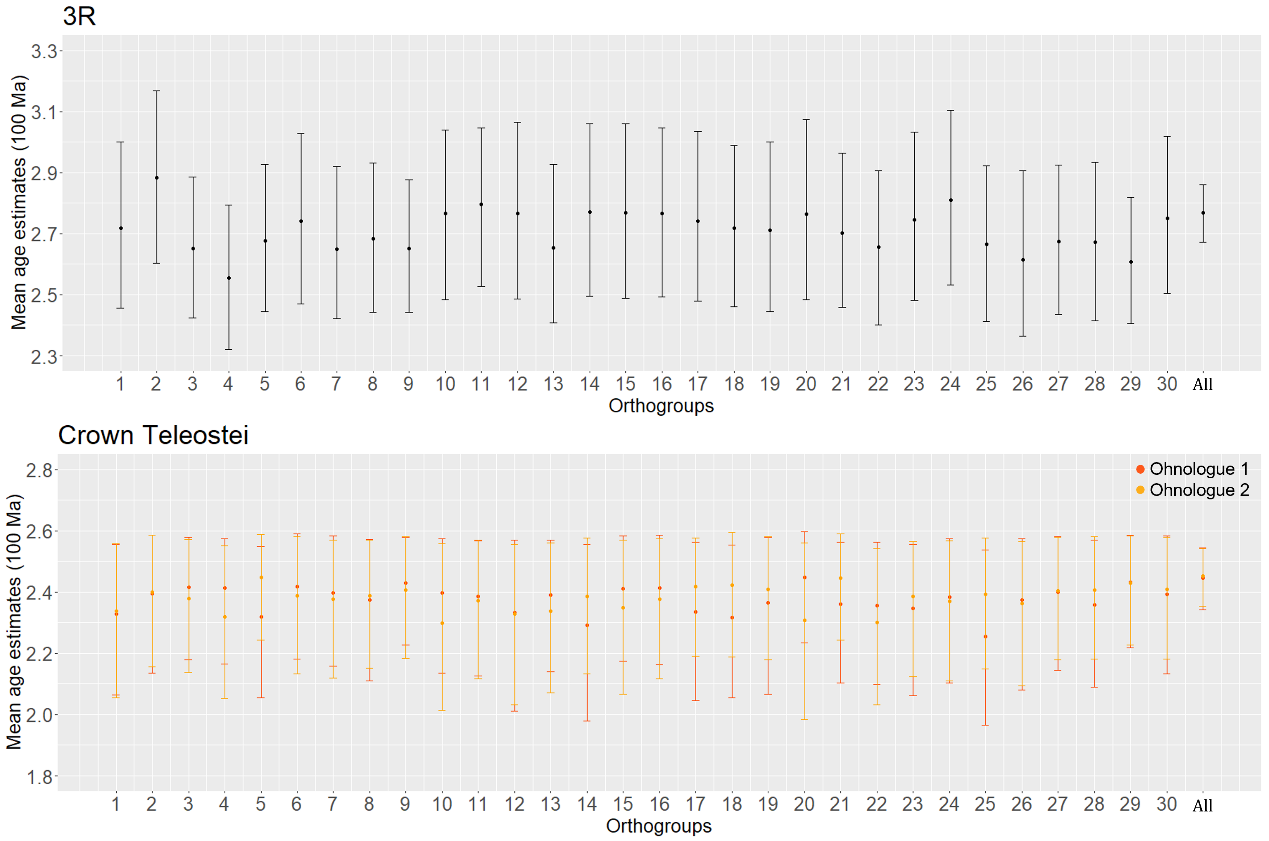

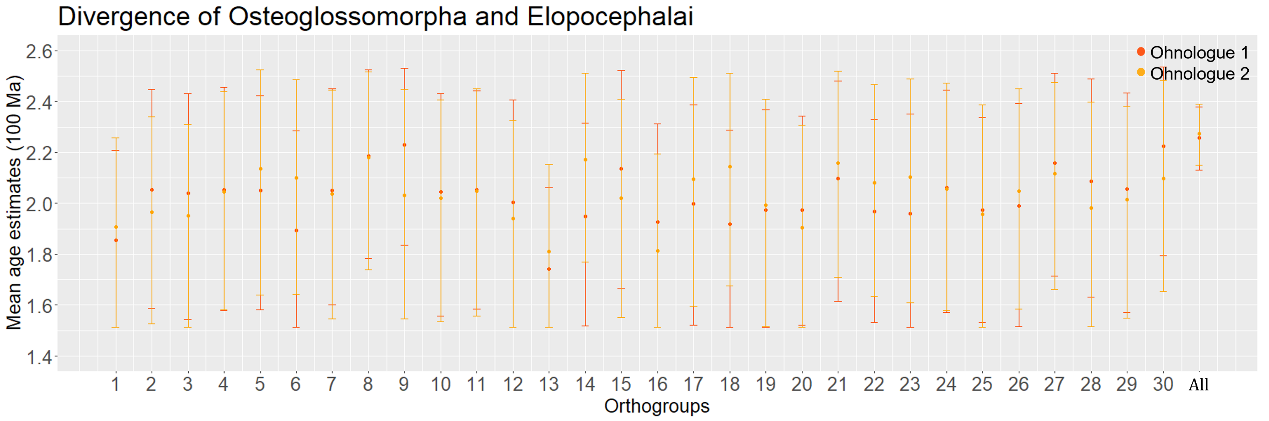

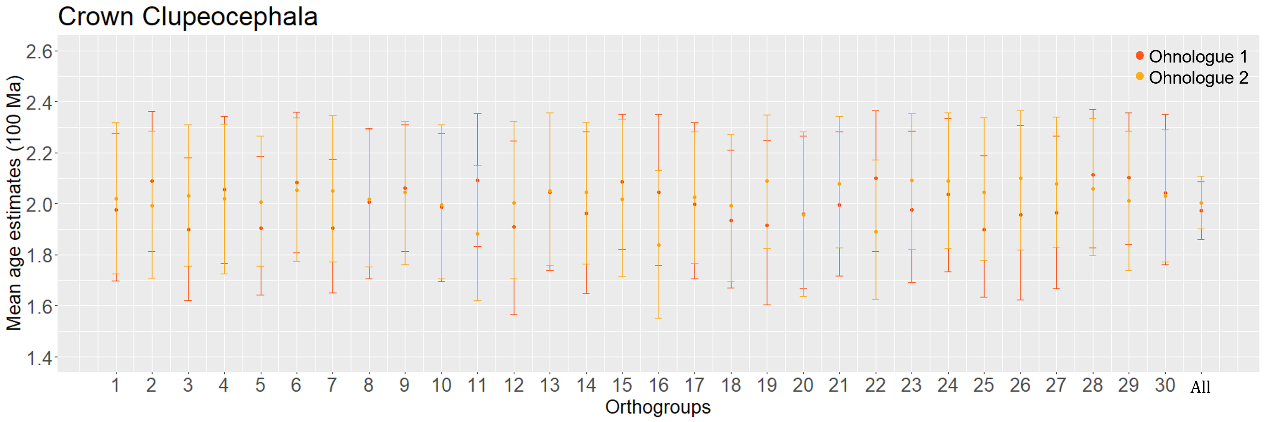
**

**
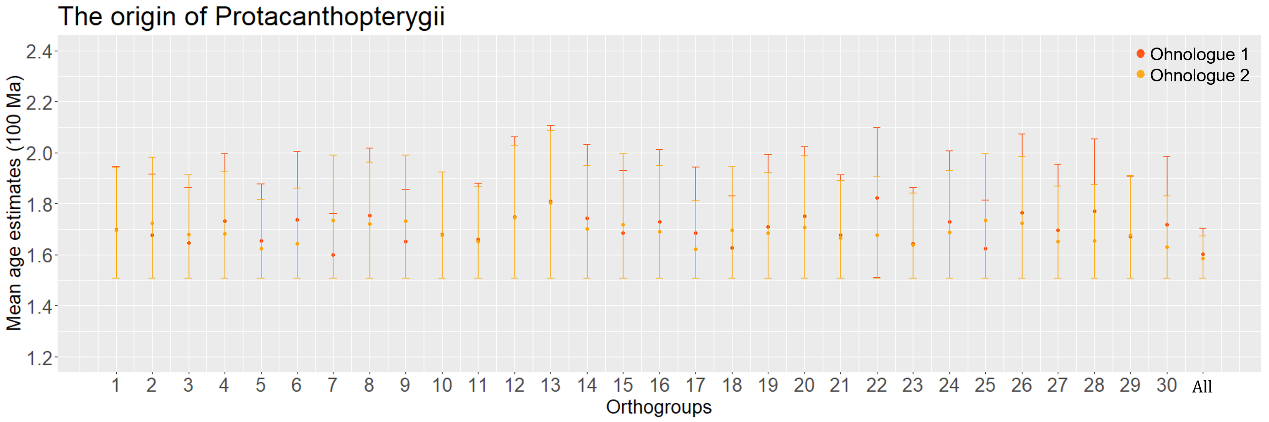

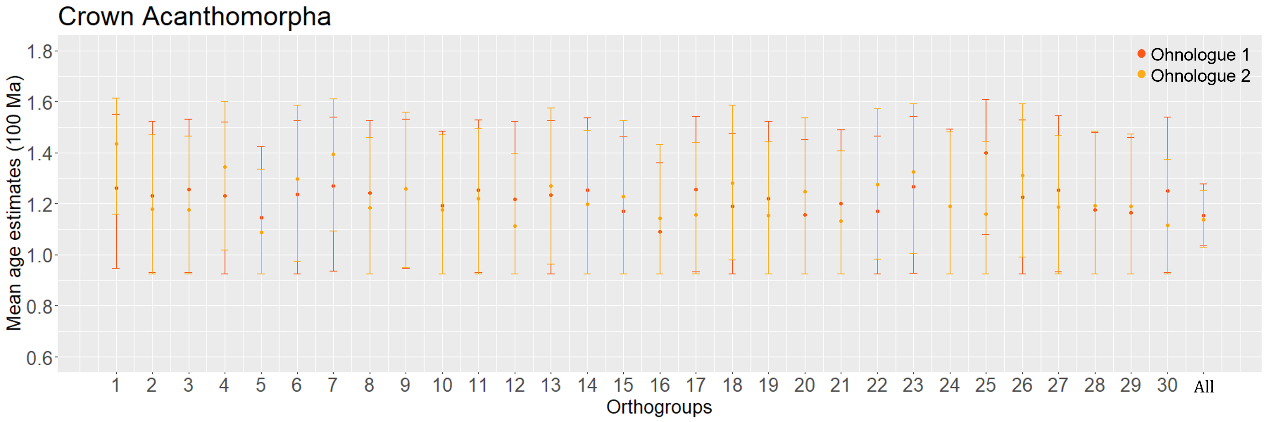

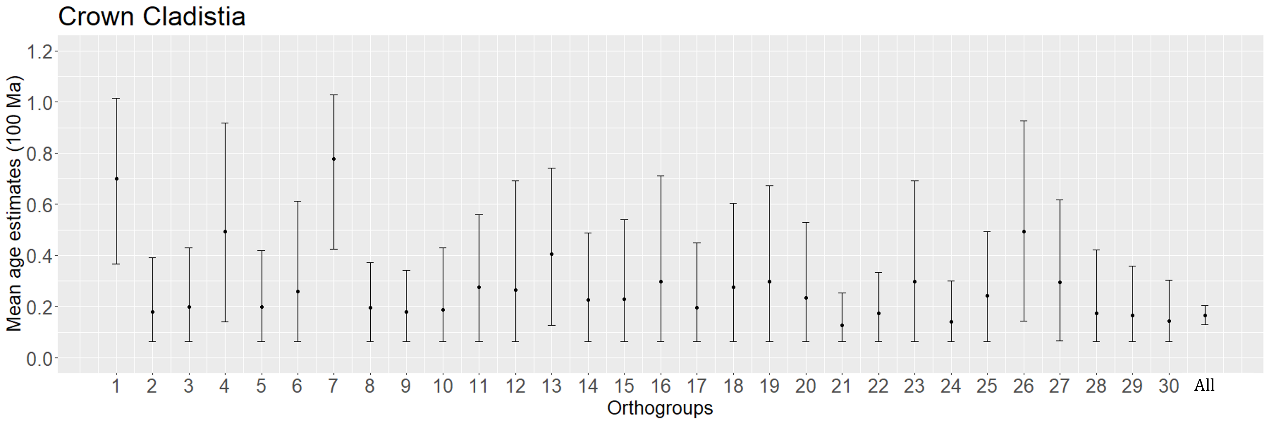

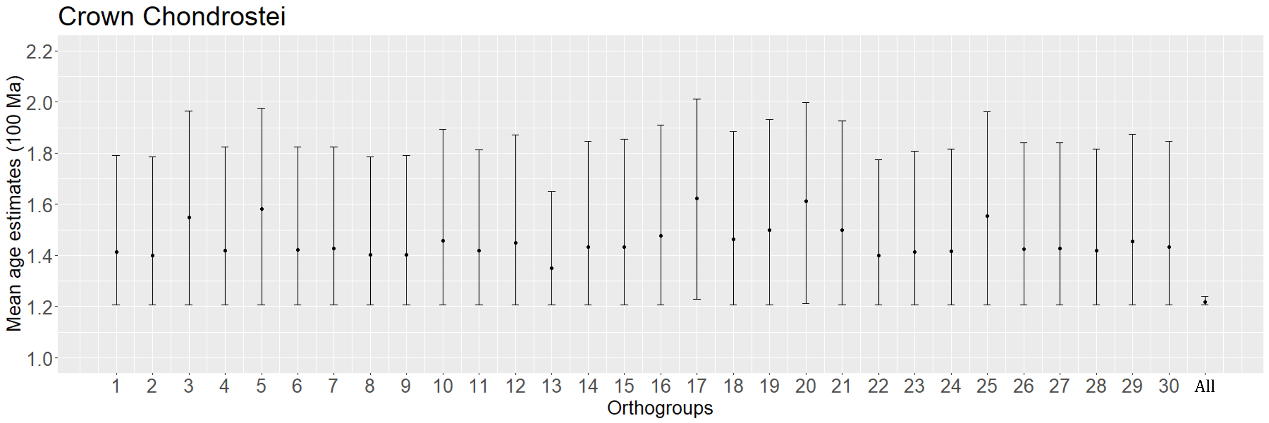

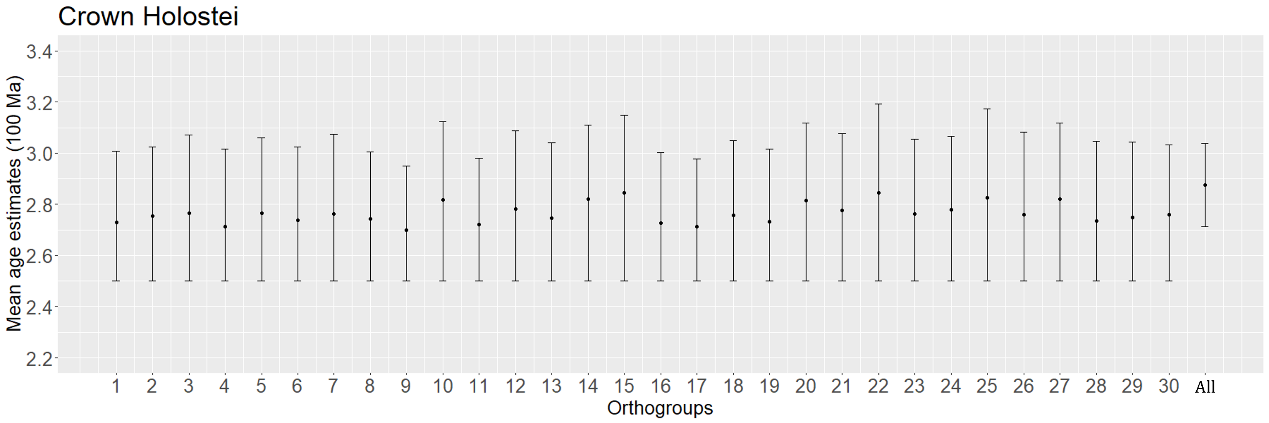
**

**Supplementary Figure 1.** Age estimates of nodes based on different orthogroups. The numbers correspond to individual orthogroups, while 'all' represents the results obtained from the concatenated dataset comprising all 30 orthogroups. The central dot within each data represents the mean age estimate, while the surrounding bars depict the 95% HPD interval of the estimate.

1. **Calibrations for dating the 3R event.**

**Node 1 | crown-Actinopterygii | Root node | Divergence of Cladistia-** **Actinopteri**

**Fossil**: Giles et al. (2017) identify *Platysomus superbus* from the Glencartholm Fauna of the Glencartholm Volcanic Group, Upper Border Group of the Calcifererous Sandstone, Glencartholm, Scotland (Moy-Thomas & Bradley-Dyne, 1938), as the oldest crown actinopterygian.

**Hard minimum:** 333.48 Ma

**Soft maximum:** 422.4 Ma

**Age justification:** the age of the Glencartholm Fauna was established by Benton et al. (2015) in association with their calibration for crown-Chondrichthyes. This is based ultimately on the age of the *Gnathodus bilineatus* conodont zone in the UK. This age of this biozone has subsequently been recalibrated and it is best constrained by a U/Pb absolute date from near the top of the succeeding *Lochriea mononodosa* biozone, as 333.87 Ma ± 0.39 Myr. Therefore, a minimum constraint on the age of crown-Actinopterygii is 333.48 Ma (Artez et al. 2020; Schmitz and Davydov, 2012).

**Node 2 | crown-Polypteriformes | Polypterus-Erpetoichthys**

**Hard Minimum**: 6.38 Ma

**Soft Maximum**: 99 Ma

Following Broughton et al. (2013), though with redating of the Anthracotheriid Unit of Toros-Menalla, Chad (from which the holotype of *Polypterus faraou* [TM090-001-039] was recovered; Otero et al. 2006) by Lebatard et al. (2008), the minimum age for which is 6.83 Ma ± 0.45 Myr, which results in a minimum constraint of 6.38 Ma.

**Node 3 | crown-Actinopteri | Chondrostei-Neopterygii**

**Fossil**: Giles et al. (2017) identify *Platysomus superbus* from the Glencartholm Fauna of the Glencartholm Volcanic Group, Upper Border Group of the Calcifererous Sandstone, Glencartholm, Scotland (Moy-Thomas & Bradley-Dyne, 1938), as the oldest crown actinopterygian, specifically, as a member of Chondrostei.

**Hard minimum:** 333.48 Ma

**Soft maximum:** 373 Ma

**Age justification:** the age of the Glencartholm Fauna was established by Benton et al. (2015) in association with their calibration for crown-Chondrichthyes. This is based ultimately on the age of the *Gnathodus bilineatus* conodont zone in the UK. This age of this biozone has subsequently been recalibrated and it is best constrained by a U/Pb absolute date from near the top of the succeeding *Lochriea mononodosa* biozone, as 333.87 Ma ± 0.39 Myr. Therefore, a minimum constraint on the age of crown-Actinopteri as 333.48 Ma (Artez et al. 2020; Schmitz and Davydov, 2012). Soft Maximum from Giles et al. (2017).

**Node 4 | crown-Chondrostei | Sturgeon-Paddlefish**

**Hard minimum:** 120.8 Ma

**Soft maximum:** 201.5 Ma

Following Benton et al. (2015).

**Node 5 | crown-Neopterygii | Holostei-Teleostei**

**Hard minimum:** 250.0 Ma

**Soft maximum:** 331.1 Ma

Following Benton et al. (2015).

**Node 6 | crown-Holostei | Halecomorphi-Ginglymodi**

**Hard minimum:** 250.0 Ma

**Soft maximum:** 331.1 Ma

Following Benton et al. (2015).

**Node 8 & Node 13 | crown-Teleostei | (Osteoglossomorpha+Elopomorpha)-Clupeocephala**

**Hard minimum:** 151.2 Ma

**Soft maximum:** 252.7 Ma

Following Benton et al. (2015).

**Node 9 & Node 14 | Elopomorpha-Osteoglossomorpha**

**Hard minimum:** 151.2 Ma

**Soft maximum:** 252.7 Ma

Following Benton et al. (2015) who used *Anaethalion zapporum* (Arratia, 2000 from the Rögling Formation, vil-lage of Schamhaupten, near Eichstätt, Bavaria, Germany; holotype JM SCH 85, Jura Museum,Eichstätt, Germany), the oldest record of Elopiformes, to calibrate the Elopiformes-Osteoglossomorpha divergence. However, in their sense, this was equivalent to crown-Teleostei; we follow Dornburg et al. (2021) in recognising the Elopiformes-Osteoglossomorpha divergence as a clade, sister to all other crown-teleosts (i.e., Otocephala plus Euteleostei).

**Node 10 & Node 15 | crown-Clupeocephala**  **| Otocephala-Euteleostei**

**Hard minimum:** 150.94 Ma

**Soft maximum:** 235 Ma

Following Benton et al. (2015).

**Node 11 & Node 16 | Protacanthopterygii-(Neoteleostei+Stomiati)**

**Fossil Taxon and Specimen.** *Tischlingerichthys viohli* Arratia, 1997 from the Mörnsheim Formation of Mühlheim, Bavaria, Germany (holotype JM Moe8, Jura Museum, Eichstätt, Germany).

**Phylogenetic Justification**. *Tischlingerichthys* is placed as the sister taxon of the ostariophysans Gordichthys and Chanos to the exclusion of clupeomorphs and other teleosts in a maximum parsi-mony analysis of morphological characters (Arratia, 1997; Arratia, 2001).

**Hard minimum:** 150.94 Ma

**Hard minimum:** 235 Ma

Following Benton et al. (2015).

**Node 12 & Node 17 | crown-Acanthomorpha | Gadiformes and Percomorpha**

Minimum Age. 92.47 Ma

Soft Maximum Age. 158.3 Ma

Following Benton et al. (2015) with a revision to the dating of the Hajula Lagerstätte (Sannine Limestone, Hajula, Lebanon) from which the minimum-informing fossil record (*Stichocentrus liratus*; holotype, NHMUK P.47835, The Natural History Museum, London, UK) is derived. Benton et al. (2015) argued for an early Cenomanian age based on records of ammonites attributed to *Mantelliceras mantelli* known from the overlying Cenomanian subdivision Va (Zummofen, 1926; Dalla Vecchia et al., 2002). However, Wippich and Lehmann (2004) call into question these unevidenced records of *M. mantelli* and their records of Allocrioceras annulatum can be attributed to the Upper Cenomanian *Sciponoceras gracile* Zone of the Western Interior Seaway. Ogg et al. (2012) provides an interpolated age of 92.47 Ma for the top of the *Sciponoceras gracile* Zone.

**Acknowledgments**: We thank Matt Friedman (U Michigan) for informing us of the revised age of the Hajula Lagerstätte.

**Reference**

Aretz, M., Herbig, H. G., Wang, X. D., Gradstein, F. M., Agterberg, F. P., and Ogg, J. G., 2020, The Carboniferous Period, Geologic Time Scale 2020, p. 811-874.

Arratia, G., 1997, Basal teleosts and teleostean phylogeny: Palaeo Ichthyologica, v. 7, p. 1-168.

Arratia, G., 2000, Remarkable teleostean fishes from the Late Jurassic of southern Germany and their phylogenetic relationships: Mitteilungen aus dem Museum für Naturkunde in Berlin, Geowissenschaftliche Reihe, v. 3, p. 137-179.

Arratia, G., 2001, The sister-group of Teleostei: consensus and disagreements: Journal of Vertebrate Paleontology, v. 21, no. 4, p. 767-773.

Benton, M. J., Donoghue, P. C. J., Asher, R. J., Friedman, M., Near, T. J., and Vinther, J., 2015, Constraints on the timescale of animal evolutionary history: Palaeontologia Electronica, v. 18, no. 1, p. 1-106.

Broughton, R. E., Betancur-R, R., Li, C., Arratia, G., and Ortí, G., 2013, Multi-locus phylogenetic analysis reveals the pattern and tempo of bony fish evolution: PLoS currents, v. 5.

Dornburg, A., and Near, T. J., 2021, The Emerging Phylogenetic Perspective on the Evolution of Actinopterygian Fishes: Annual Review of Ecology, Evolution, and Systematics, v. 52, no. 1, p. 427-452.

Giles, S., Xu, G. H., Near, T. J., and Friedman, M., 2017, Early members of 'living fossil' lineage imply later origin of modern ray-finned fishes: Nature, v. 549, no. 7671, p. 265-268.

Lebatard, A. E., Bourles, D. L., Duringer, P., Jolivet, M., Braucher, R., Carcaillet, J., Schuster, M., Arnaud, N., Monie, P., Lihoreau, F., Likius, A., Mackaye, H. T., Vignaud, P., and Brunet, M., 2008, Cosmogenic nuclide dating of Sahelanthropus tchadensis and Australopithecus bahrelghazali: Mio-Pliocene hominids from Chad: Proc Natl Acad Sci U S A, v. 105, no. 9, p. 3226-3231.

Moy-Thomas, J., and Dyne, M. B., 1938, XVII.—The Actinopterygian Fishes from the Lower Carboniferous of Glencartholm, Eskdale, Dumfriesshire: Earth and Environmental Science Transactions of The Royal Society of Edinburgh, v. 59, no. 2, p. 437-480.

Near, T. J., Dornburg, A., Tokita, M., Suzuki, D., Brandley, M. C., and Friedman, M., 2014, Boom and bust: ancient and recent diversification in bichirs (Polypteridae: Actinopterygii), a relictual lineage of ray-finned fishes: Evolution, v. 68, no. 4, p. 1014-1026.

Ogg, J. G., and Hinnov, L. A., 2012, Cretaceous, in Gradstein, F. M., Ogg, J. G., Schmitz, M., and Ogg, G., eds., The geologic time scale 2012, Volume 2, Elsevier, p. 793-853.

Otero, O., Likius, A., Vignaud, P., and Brunet, M., 2006, A new polypterid fish: Polypterus faraou sp. nov.(Cladistia, Polypteridae) from the Late Miocene, Toros-Menalla, Chad: Zoological Journal of the Linnean Society, v. 146, no. 2, p. 227-237.

Schmitz, M. D., and Davydov, V. I., 2012, Quantitative radiometric and biostratigraphic calibration of the Pennsylvanian–Early Permian (Cisuralian) time scale and pan-Euramerican chronostratigraphic correlation: GSA Bulletin, v. 124, no. 3-4, p. 549-577.

Wippich, M. G. E., and Lehmann, J., 2004, Allocrioceras from the Cenomanian (mid‐Cretaceous) of the Lebanon and its bearing on the palaeobiological interpretation of heteromorphic ammonites: Palaeontology, v. 47, p. 1093-1107.
