# Supplementary material for: Molecular Dating of the Teleost Whole Genome Duplication (3R) Is Compatible With the Expectations of Delayed Rediploidization
Source: Genome Biol Evol. 2024 Jun 24;16(7):evae128. doi: 10.1093/gbe/evae128 (PMC11259977; doi:10.1093/gbe/evae128)
Supplement: evae128_Supplementary_Data [file evae128_supplementary_data.zip › 4_Supplementary_table_6_synteny.pdf]

Zebrafishs' genome have 25 pairs of chromosomes

Ohnologue pairs		Name	ID	From which chromosome	KEGG Number	Gene name on NCBI	Ensembl ID	Chromosome Number in GenomicusFish	Location on the Chromosome	The unique identifier of the post-duplication gene family from the comparative atlas downloaded from (Parey 2022)	Predicted post-duplication ancestral chromosome
1	1	serine incorporator 2	XP_009290780.1	chromosome="16"	K24176	serinc2l	ENSDARG00000053425	16	34,459,066 - 34,477,805	ancGene_TGD_9160_B	2b
	2	unnamed protein product [Danio rerio](serine incorporator 2 precursor from previous blast)	NP_001038647.1	chromosome="19"	Not match	serinc2	ENSDARG00000056532	19	15,417,372 - 15,420,678	ancGene_TGD_9160_A	2a
2	1	lysyl oxidase homolog 2b precursor [Danio rerio]	NM_001080626.1	chromosome="5"	K00280	loxl2b	ENSDARG00000044074	5	27,440,335 - 27,487,789	ancGene_TGD_5229_B	9a
	2	lysyl oxidase homolog 2A precursor [Danio rerio]	NP_001092714.1	chromosome="10"	K00280	loxl2a	ENSDARG00000044010	10	20,407,888 - 20,445,549	ancGene_TGD_5229_A	9b
3	1	neuronal acetylcholine receptor subunit alpha-9-II-like [Danio rerio]	XP_001338964.6	chromosome="14"	Not match	LOC798522	ENSDARG00000099181	14	669,982 - 677,801	ancGene_TGD_25139_B	6b
	2	neuronal acetylcholine receptor subunit alpha-9 isoform X2	XP_021326868.1	chromosome="1"	K04810	chrna9	ENSDARG00000054680	1	23,408,622 - 23,417,911	ancGene_TGD_25139_A	6a
4	1	C-C chemokine receptor type 6 [Danio rerio]	XP_002665313.2	chromosome="17"	K04181	ccr6b	ENSDARG00000038968	17	51,642,687 - 51,651,631	ancGene_TGD_8954_A	1a
	2	C-C chemokine receptor type 6a [Danio rerio]	NP_001093461.1	chromosome="20"	K04181	ccr6a	ENSDARG00000087474	20	30,610,547 - 30,614,519	ancGene_TGD_8954_A	1a
5	1	uncharacterized protein LOC566223 [Danio rerio]	NP_001122203	chromosome="5"	no KO assigned	atf5a	ENSDARG00000068096	5	30,414,250 - 30,418,636	ancGene_TGD_1995_B	8b
	2	uncharacterized protein atf5b isoform X1 [Danio rerio]	XP_021322033.1	chromosome="15"	K09044	atf5b	ENSDARG00000077785	15	17,868,292 - 17,868,870	ancGene_TGD_1995_A	8a
6	1	CLOCK-interacting pacemaker a [Danio rerio]	NP_001314832	chromosome="17"	no KO assigned	cipca	ENSDARG00000075397	17	44,756,247 - 44,761,580	ancGene_TGD_14045_B	1b
	2	CLOCK-interacting pacemaker-like [Danio rerio]	XP_005170287.1	chromosome="20"	no KO assigned	cipcb	ENSDARG00000078095	20	54,336,137 - 54,340,428	ancGene_TGD_14045_A	1a
7	1	brain-specific angiogenesis inhibitor 1-associated protein 2-like protein 2 isoform X1 [Danio rerio]	XP_005161865.1	chromosome="22"	K23645	baiap2l2b	ENSDARG00000060933	22	29,135,846 - 29,166,693	ancGene_TGD_29834_B	5b
	2	brain-specific angiogenesis inhibitor 1-associated protein 2-like protein 2 isoform X2 [Danio rerio]	XP_009297664.1	chromosome="3"	K23645	baiap2l2a	ENSDARG00000016483	3	24,448,102 - 24,458,281	ancGene_TGD_29834_A	5a
8	1	dipeptidyl peptidase 4 [Danio rerio]	XP_001920834.4	chromosome="9"	no KO assigned	fap	ENSDARG00000078468	9	51,225,345 - 51,261,339	ancGene_TGD_35811_B	3a
	2	dipeptidyl peptidase 4 [Danio rerio]	NP_001154809.1	chromosome="22"	K01278	dpp4	ENSDARG00000079420	22	11,628,438 - 11,648,094	ancGene_TGD_35811_A	3b
9	1	NADPH oxidase organizer 1b [Danio rerio]	NP_001313627	chromosome="12"	No match	p47_phox_C	ENSDARG00000056374	12	18,445,604 - 18,454,422	ancGene_TGD_2474_B	5b
	2	NADPH oxidase organizer 1a [Danio rerio]	NP_001071052.1	hromosome="3"	K17934	noxo1a	ENSDARG00000041294	3	62,161,184 - 62,168,286	ancGene_TGD_2474_A	5a
10	1	alanine--glyoxylate and serine--pyruvate aminotransferase a [Danio rerio]	NP_001002331.1	chromosome="6"	K00830	agxta	ENSDARG00000052099	6	27,112,884 - 27,123,327	ancGene_TGD_5187_A	13a
	2	alanine--glyoxylate and serine--pyruvate aminotransferase b [Danio rerio]	NP_998327.1	chromosome="2"	K00830	agxtb	ENSDARG00000018478	**2**	22,677,015 - 22,688,651	ancGene_TGD_5186_A	13b
11	1	potassium voltage-gated channel subfamily A member 10-like [Danio rerio]	XP_002667319.1	chromosome="23"	K04881	kcna10a	NO_MATCHING				
	2	potassium voltage-gated channel subfamily A member 10-like [Danio rerio]	XP_021332962.1	chromosome="6"	No match	LOC567521	NO_MATCHING				
12	1	somatostatin receptor type 2-like [Danio rerio]	XP_017210358.1	chromosome="3"	K04218	sstr2b	ENSDARG00000069806	3	62,416,556 - 62,417,677	ancGene_TGD_34656_A	5a
	2	somatostatin receptor type 2-like [Danio rerio]	XP_005170178.1	chromosome="12"	K04218	sstr2a	ENSDARG00000059090	12	1,556,147 - 1,557,286	ancGene_TGD_34656_B	5b
13	1	synaptophysin-like 2b isoform X1 [Danio rerio]	XP_017212875.1	chromosome="8"	no KO assigned	sypl2b	ENSDARG00000000690	8	25,079,470 - 25,090,458	ancGene_TGD_16223_B	12a
	2	synaptophysin-like protein 2a [Danio rerio]	NP_956961.2	chromosome="11"	no KO assigned	sypl2a	ENSDARG00000055307	11	36,355,348 - 36,376,073	ancGene_TGD_16223_A	12b
14	1	unconventional myosin-VIIb-like isoform X1 [Danio rerio]	XP_021327440.1	chromosome="6"	K21868	LOC108190403/MYO7B	ENSDARG00000044441	6	28,054,639 - 28,111,306	ancGene_TGD_22049_A	13a
	2	unconventional myosin-VIIa isoform X2 [Danio rerio]	XP_021322881	chromosome="2"	K21868	myo7bb	ENSDARG00000077201	**2**	22,880,993 - 22,927,581	ancGene_TGD_22048_A	13b
15	1	espin-like protein [Danio rerio]	XP_017207109.1	chromosome="2"	K24047	espnlb	ENSDARG00000093112	**2**	22,765,492 - 22,792,460	NO_MATCH	NO_MATCH
	2	espin-like protein [Danio rerio]	XP_021332809.1	chromosome="6"	K24047	espnla	ENSDARG00000078211	6	27,315,173 - 27,338,044	ancGene_TGD_8567_B	13a
16	1	elongation of very long chain fatty acids protein 4a [Danio rerio]	NP_957090.1	chromosome="16"	K10249	elovl4a	ENSDARG00000006773	16	5,156,420 - 5,167,280	ancGene_TGD_26505_B	2b
	2	elongation of very long chain fatty acids protein 4b [Danio rerio]	NP_956266.1	chromosome="23"	K10249	elovl4b	ENSDARG00000027495	23	40,410,644 - 40,438,581	ancGene_TGD_26505_A	1a
17	1	P2Y purinoceptor 3-like [Danio rerio]	XP_021333479.1	chromosome="7"	No match	si:dkey-6n21.13	ENSDARG00000069459	7	26,058,772 - 26,060,908	ancGene_TGD_6594_A	8a
	2	P2Y purinoceptor 3-like [Danio rerio]	XP_009303554	chromosome="10"	No match	LOC100538037	NO_MATCHING				
18	1	lipase member H-like [Danio rerio]	XP_001342691.1	chromosome="24"	K19404	lipib	ENSDARG00000005332	24	24,086,491 - 24,101,132	ancGene_TGD_26011_A	13a
	2	lipase member H precursor [Danio rerio]	NP_001003499.1	chromosome="21"	K19404	lipia	ENSDARG00000007108	21	34,947,738 - 34,951,265	ancGene_TGD_26011_B	13b
19	1	zinc finger protein GLIS2 isoform X1 [Danio rerio]	XP_009294691.1	chromosome="22"	K09233	glis2a	ENSDARG00000078388	22	26,524,760 - 26,558,166	ancGene_TGD_3643_A	5b
	2	zinc finger protein GLIS2 isoform X1 [Danio rerio]	XP_021324946.1	chromosome="3"	K09233	glis2b	ENSDARG00000100232	3	12,334,509 - 12,335,927	ancGene_TGD_3643_B	5a
20	1	P2X purinoceptor 3 [Danio rerio]	NP_571698.2	chromosome="14"	K05217	p2rx3a	ENSDARG00000010477	14	21,942,383 - 21,959,712	ancGene_TGD_36118_B	6b
	2	P2X purinoceptor 3b [Danio rerio]	NP_945337.3	chromosome="1"	K05217	p2rx3b	ENSDARG00000029718	1	44,746,154 - 44,758,388	ancGene_TGD_36118_A	6a
21	1	leucine-rich repeat, immunoglobulin-like domain and transmembrane domain-containing protein 1b prec	NP_001036218.1	chromosome="12"	K24491	lrit1b	ENSDARG00000099406	12	48,972,915 - 48,976,282	ancGene_TGD_7048_B	4b
	2	leucine-rich repeat, immunoglobulin-like domain and transmembrane domain-containing protein 1a prec	NP_001018174.2	chromosome="13"	K24491	lrit1a	ENSDARG00000019179	13	29,462,249 - 29,467,558	ancGene_TGD_7048_A	4a
22	1	coagulation factor IXb precursor [Danio rerio]	NP_001035400.1	chromosome="10"	K01321	f9b	ENSDARG00000029493	10	26,747,755 - 26,757,486	ancGene_TGD_21993_B	7b
	2	coagulation factor IXa precursor [Danio rerio]	NP_878288.2	chromosome="14"	K01321	f9a	ENSDARG00000010097	14	32,431,032 - 32,437,728	ancGene_TGD_21993_A	7a
23	1	insulin-like growth factor-binding protein 1a precursor [Danio rerio]	NP_775390.3	chromosome="20"	K23578	igfbp1a	ENSDARG00000099351	20	6,808,774 - 6,812,688	ancGene_TGD_21577_B	13a
	2	insulin-like growth factor-binding protein 1b precursor [Danio rerio]	NP_001091727.1	chromosome="2"	K23578	igfbp1b	ENSDARG00000038666	2	124,433 - 127,945	ancGene_TGD_21577_A	13b
24	1	protein Z-dependent protease inhibitor [Danio rerio]	XP_009291625.1	chromosome="17"	K04525	si:ch1073-416d2.3	ENSDARG00000036968	17	53,294,228 - 53,295,507	ancGene_TGD_23870_B	1b
	2	serpin peptidase inhibitor, clade A (alpha-1 antiproteinase, antitrypsin), member 10a precursor [Danio reric	NP_001038536	chromosome="20"	K04525	serpina10a	ENSDARG00000005924	20	27,371,394 - 27,381,691	ancGene_TGD_23870_A	1a
25	1	ceramide synthase 2-like [Danio rerio]	XP_693668.1	chromosome="16"	K24621	cers2b	ENSDARG00000058992	16	1,383,914 - 1,399,328	ancGene_TGD_17238_B	2b
	2	ceramide synthase 2a [Danio rerio]	NP_705957.1	chromosome="19"	K24621	cers2a	ENSDARG00000013704	19	8,775,453 - 8,798,178	ancGene_TGD_17238_A	2a
26	1	tetranectin [Danio rerio]	XP_021322479.1	chromosome="16"	K17520	clec3ba	ENSDARG00000076541	16	2,843,428 - 2,844,195	ancGene_TGD_16633_A	2b
	2	tetranectin-like [Danio rerio]	XP_003200569.1	chromosome="19"	K17520	clec3bb	ENSDARG00000099500	19	2,857,714 - 2,861,444	ancGene_TGD_16633_B	2a
27	1	T-lymphoma invasion and metastasis-inducing protein 1 isoform X1 [Danio rerio]	XP_009290217.1	chromosome="15"	K05731	tiam1b	ENSDARG00000025920	15	42,431,198 - 42,551,156	ancGene_TGD_25056_A	8a
	2	T-lymphoma invasion and metastasis-inducing protein 1 [Danio rerio]	XP_001924044.3	chromosome="10"	K05731	tiam1a	ENSDARG00000078430	10	25,581,941 - 25,591,194	ancGene_TGD_25056_B	8b
28	1	calcium-activated potassium channel subunit beta-2-like isoform X3 [Danio rerio]	XP_021335408.1	chromosome="11"	K04938	si:ch211-247n2.1	ENSDARG00000001712	11	8,303,941 - 8,320,868	ancGene_TGD_36100_A	13a
	2	calcium-activated potassium channel subunit beta-2 [Danio rerio]	NP_001013543.1	chromosome="2"	No match	kcnmb2a	ENSDARG00000006568	2	7,818,368 - 7,829,400	ancGene_TGD_36100_B	13b
29	1	contactin-associated protein-like 2a precursor [Danio rerio]	NP_001268920.1	chromosome="24"	K07380	cntnap2a	ENSDARG00000058969	24	17,420,803 - 17,444,067	ancGene_TGD_21230_B	13a
	2	contactin-associated protein-like 2b precursor [Danio rerio]	NP_001289171	chromosome="2"	K07380	cntnap2b	ENSDARG00000074558	2	50,276,755 - 50,298,337	ancGene_TGD_21230_A	13b
30	1	contactin-1a precursor [Danio rerio]	NP_851300.2	chromosome="25"	K06759	cntn1a	ENSDARG00000087843	25	126,174 - 137,524	ancGene_TGD_25444_A	11b
	2	contactin 1b precursor [Danio rerio]	NP_001014814.1	chromosome="4"	K06759	si:dkey-240a12.2	ENSDARG00000045685	4	13,733,838 - 13,756,690	ancGene_TGD_25444_B	11a
